# Myocyte membrane and microdomain modifications in diabetes: determinants of ischemic tolerance and cardioprotection

**DOI:** 10.1186/s12933-017-0638-z

**Published:** 2017-12-04

**Authors:** Jake Russell, Eugene F. Du Toit, Jason N. Peart, Hemal H. Patel, John P. Headrick

**Affiliations:** 1 Menzies Health Institute Queensland, Griffith University, Southport, QLD Australia; 2 VA San Diego Healthcare System and Department of Anesthesiology, University of California San Diego, San Diego, USA; 3 School of Medical Science, Griffith University, Southport, QLD 4217 Australia

**Keywords:** Caveolae, Cardioprotection, Cholesterol, Diabetes, Fatty acids, Glucose transport, Infarction, Phospholipids

## Abstract

Cardiovascular disease, predominantly ischemic heart disease (IHD), is the leading cause of death in diabetes mellitus (DM). In addition to eliciting cardiomyopathy, DM induces a ‘wicked triumvirate’: (i) increasing the risk and incidence of IHD and myocardial ischemia; (ii) decreasing myocardial tolerance to ischemia–reperfusion (I–R) injury; and (iii) inhibiting or eliminating responses to cardioprotective stimuli. Changes in ischemic tolerance and cardioprotective signaling may contribute to substantially higher mortality and morbidity following ischemic insult in DM patients. Among the diverse mechanisms implicated in diabetic impairment of ischemic tolerance and cardioprotection, changes in sarcolemmal makeup may play an overarching role and are considered in detail in the current review. Observations predominantly in animal models reveal DM-dependent changes in membrane lipid composition (cholesterol and triglyceride accumulation, fatty acid saturation vs. reduced desaturation, phospholipid remodeling) that contribute to modulation of caveolar domains, gap junctions and T-tubules. These modifications influence sarcolemmal biophysical properties, receptor and phospholipid signaling, ion channel and transporter functions, contributing to contractile and electrophysiological dysfunction, cardiomyopathy, ischemic intolerance and suppression of protective signaling. A better understanding of these sarcolemmal abnormalities in types I and II DM (T1DM, T2DM) can inform approaches to limiting cardiomyopathy, associated IHD and their consequences. Key knowledge gaps include details of sarcolemmal changes in models of T2DM, temporal patterns of lipid, microdomain and T-tubule changes during disease development, and the precise impacts of these diverse sarcolemmal modifications. Importantly, exercise, dietary, pharmacological and gene approaches have potential for improving sarcolemmal makeup, and thus myocyte function and stress-resistance in this ubiquitous metabolic disorder.

## Diabetes impacts myocardial ischemic tolerance and cardioprotection

Clinical evidence indicates DM sensitizes human hearts to I–R injury [1, 2], which is generally consistent with experimental findings in animal models, though conflicting observations arise. Compounding the problem of infarct intolerance, DM may also render hearts broadly refractory to established cardioprotective stimuli that include ischemic pre- and post-conditioning (direct or remote) and protective G protein-coupled receptor (GPCR) agonism, together with the anti-infarct effects of ATP-gated K^+^ channel (K_ATP_) openers, anesthetics, phosphodiesterase-5 (PDE-5) inhibition and heat shock activation [3–6]. Thus, while elusive cardioprotective therapies [6–8] are of particular value in the high-risk DM population, implementation appears an even greater challenge in this cohort. Prevalence of DM and insulin-resistance in those suffering IHD may in turn contribute to poor translation of experimental cardioprotection in these patients. Relatively few studies specifically address the conundrum of I–R sensitivity and cardioprotective insensitivity in DM [6]. Investigations to date implicate a diversity of mechanisms extending beyond fundamental alterations in glucose and lipid metabolism, including associated glycation/glycosylation [9], oxidative stress [10, 11], abnormal survival kinase signaling [12–14] and exosome dysfunction [15], excessive ubiquitin–proteasome system activity [16], suppression of sirtuin-1 expression [17], and changes in miRNA expression [18], among others. Considerable attention has focused on mitochondrial dysfunction, including shifts in quality control mechanisms (mitophagy, fission/fusion), as a point of convergence in the complex pathogenesis of diabetic cardiomyopathy [10, 19, 20]. However, the sarcolemma is also a critical though under-appreciated nexus, influencing DM progression and its impacts [21]. Indeed, transcriptomic profiling indicates that the largest group of diabetes-modified cardiac genes encode membrane/plasma membrane components [22], consistent with more recent studies identifying DM-dependent changes in transcripts for membrane and structural proteins, sarcolemmal receptors and ion channels [23]. Transporters for glucose and fatty acids, ion channels and exchangers, and receptor systems governing insulin responses, inflammation, mitochondrial quality control, and cell stress, growth and death are all located within the sarcolemma, while mitochondrial function is also sensitive to sarcolemmal domains and proteins. Perturbations in membrane composition and architecture may thus be critical to the dysfunctional stress responses characteristic of diabetic myocardium, together with other cardiac outcomes including hypertrophy and contractile dysfunction. We herein review the clinical and experimental evidence of DM-dependent changes in myocardial ischemic tolerance and cardioprotection, before focusing specifically on sarcolemmal changes and their contribution to the cardiac sequelae of DM.

### Effects of DM in human myocardium

Diabetes induces a spectrum of abnormalities within the myocardium and coronary vasculature. Diastolic dysfunction, fibrosis and hypertrophy functionally and structurally underpin diabetic cardiomyopathy [10]. These changes are linked to reactive oxygen species (ROS) generation, inflammation, mitochondrial dysfunction, and abnormalities in molecular quality control, including autophagy, fission/fusion, endoplasmic reticulum (ER) stress and unfolded protein responses. Coronary endothelial dysfunction and vascular remodeling exaggerate atherosclerosis and impair vascular control and coronary perfusion, potentially contributing to cardiac dysfunction. These changes in myocardial and coronary phenotypes (and underlying molecular mechanisms) may participate in impairment of myocardial stress tolerance, hormesis and protective signaling, which may in turn further exacerbate these phenotypic changes.

#### Myocardial ischemic tolerance

The impacts of DM on myocardial ischemic tolerance and infarction remain somewhat contentious. Certainly DM worsens long-term outcomes from ischemic insult, including increased incidence of heart failure and all-cause mortality [24–26]. There is some evidence these poor outcomes may involve diabetic impairment of myocardial reperfusion [27, 28], consistent with vascular dysfunction and reduced coronary reserve [29–31]. The contribution of worsened infarction to poor post-ischemic prognosis awaits further clarification, with some contrasting data acquired. Diabetes can significantly increase infarct size as assessed via scintigraphy [1, 32] and magnetic resonance imaging (MRI) [33]. Insulin-treated DM patients also exhibit worsened myocardial infarction, mortality, major adverse cardiac events and thrombosis compared with untreated or non-DM subjects, potentially reflecting negative impacts of more complex and prolonged disease [34–36]. On the other hand, some myocardial scintigraphic [37] and MRI analyses [38, 39] report no significant differences in infarction in DM vs. non-DM STEMI patients post angioplasty.

Other evidence strongly supports exaggerated myocardial damage and cell death in DM patients: DM markedly increases morbidity and mortality (up to 90%) following cardioplegic arrest [40–42]; DM promotes pro-apoptotic signaling, apoptosis and contractile dysfunction in reperfused human myocardium [43–45]; and DM exaggerates oxidative damage and anti-oxidant depletion [45, 46], transcriptional changes and pro-inflammatory signaling [45, 47]. Analysis of I–R injury in ex vivo tissue reveals significantly impaired resistance of myocardium from T1 and T2DM patients, including increased apoptosis (partially caspase- and PARP-dependent) and oncosis [48]. Anderson et al. [49] more recently provided evidence that myocardium from DM patients has a greater propensity for mitochondria-dependent cell death. There is also evidence of exaggeration of post-ischemic contractile dysfunction in DM: the studies of Hoogslag et al. [50] and Dimitriu-Leen et al. [51] reveal worsened myocardial longitudinal strain independently of infarct size, supporting greater mechanical disruption in DM. Hyperglycemia itself has been shown to increase infarct size and mortality in infarct patients [1, 28, 52–54]. This may also involve impaired reperfusion, though there is evidence hyperglycemia exaggerates infarction by increasing the area at risk [55]. Use of insulin and sulfonylureas to manage hyperglycemia may additionally worsen ischemic injury, morbidity and mortality [34–36, 56, 57].

Conversely, there is some limited evidence myocyte ischemic tolerance might be increased in T1DM patients, though for skeletal and not cardiac tissue [58]. This is consistent with some rodent studies in acute T1DM models (see below). Nonetheless, the weight of experimental evidence supports reduced myocardial I–R tolerance in DM, encompassing exaggerated apoptosis, oncosis and infarction, contractile dysfunction and markers of oxidative damage. It remains unclear to what extent poor post-ischemic prognosis reflects exaggerated ischemic insult, impaired reperfusion, and increased propensity to cell death. The roles of individual metabolic disturbances (hyperglycemia, hyperinsulinemia, insulin-resistance, dyslipidemia), coronary dysfunction and compromised reflow, together with intrinsic myocardial stress-resistance, thus require further detailed analysis.

#### Cardioprotection

Studies broadly support the desensitization or elimination of diverse cardioprotective responses in DM myocardium, though again this is not universal. There are relatively few studies of diabetic impacts on cardioprotective responses in human myocardium. Ishihara et al. [59] reported that DM inhibits ischemic preconditioning in anterior wall infarct patients, while Lee et al. [4] present evidence of impaired preconditioning responses in DM patients undergoing angioplasty. Galiñanes and colleagues found that ex vivo myocardium from DM patients was insensitive to ischemic preconditioning [3], and subsequently identified loss of responsiveness not only to preconditioning but to phenylephrine, adenosine and diazoxide (implicating signal dysfunction proximal to protein kinase C (PKC) and p38 mitogen-activated protein kinase (MAPK) [5]. More recent studies support desensitization of DM myocardium to hypoxic preconditioning in association with impaired phosphatidylinositol 3 kinase (PI3K) and Akt signaling [60], and failure of ischemic preconditioning in myocardium from DM patients [61]. On the other hand, some studies confirm protective efficacies of anesthetic post-conditioning in ex vivo myocardium [62, 63] and of ischemic preconditioning in vivo [64] in DM patients. Additionally, a meta-analysis assessing influences of risk factor across ten trials of post-conditioning in STEMI [65] verified significant interactions with age and sex (reduced efficacy in older and/or female patients) yet not with DM. The authors concede analytical limitations may lead to an under-estimation of the influences of co-morbidities such as DM. A subsequent focused albeit smaller analysis also failed to identify interaction between DM and post-conditioning in STEMI patients [66], though also failed to detect the sex and age effects revealed by Zhou et al., highlighting limited power to detect effects in small sample sizes via posteriori statistical analysis.

#### Complicating effects of anti-hyperglycemia therapies

In addition to the underlying DM cardio-pathology, there is evidence clinical approaches to managing hyperglycemia may impair cardioprotective signaling and worsen ischemic outcomes. Sulfonylurea use is associated with greater ischemic injury and infarction in DM [56, 57], and inhibition of ischemic preconditioning in both non-DM and DM patients [67, 68] and ex vivo myocardium from DM patients [69]. Glinide also impairs preconditioning in DM patients [70, 71]. These negative impacts are consistent with their ability to inhibit K_ATP_ channels implicated in transducing or mediating cardiac protection [72].

In addition, insulin treatment has been linked to a paradoxic worsening of complications, all-cause mortality and cardiac outcomes in DM [34, 35]. Concern regarding potentially untoward effects of glycemic control arose from epidemiological evidence of increased mortality in insulin-treated vs. untreated T2DM patients [73, 74], together with observations of insulin effects on cardiac events [75, 76] and mortality in heart failure complicated by T2DM [77]. Evidence of worsened outcomes with insulin and sulfonylureas over metformin [34] suggests direct insulin- and K_ATP_ channel dependent actions rather than simple glucose-lowering. However, whether involving direct effects of insulin, influences of acutely reduced glucose (or overt hypoglycemia) following chronic hyperglycemia, or the fact insulin-treated patients often exhibit greater comorbidities and suffer more protracted disease, awaits further clarification. There are potential mechanisms by which insulin might worsen cardiovascular outcomes despite normalization of glucose. For example, insulin can induce weight gain which can exaggerate cardiovascular (and also cancer) risks, while atherogenic and mitogenic effects may accelerate atherosclerosis/IHD. Moreover, there is evidence insulin treatment up-regulates pro-inflammatory tumor necrosis factor α and interleukin-1 to a greater extent in T1DM vs. healthy animals [78], and insulin-dependent NO generation may promote oxidative stress [79], together with vascular damage through increased circulatory pulsatility [80]. Hypoglycemia as a result of poor glycemic control may also increase arrhythmogenesis, cardiac events and mortality [81], though whether this reflects a causal relationship is unclear, with other studies reporting no association between hypoglycemia and cardiac or all-cause mortality in T2DM [82]. Conversely, there is evidence hyperglycemia can promote compensatory mechanisms that protect against I–R injury, including improvements in anti- vs. pro-oxidant balance and protein integrity [83], which might be countered by reductions in glucose levels. The hearts of diabetic patients do appear desensitized to the injurious effects of elevated glucose [38]. However, further work is needed in disentangling these complexities.

### Effects of DM in animal and in vitro models

There are some conflicting reports regarding impacts of DM on myocardial infarction and cardioprotection in animal models. Reviewed previously [2, 84, 85], studies in different species and models report increases, no change, or reductions in infarct size with DM. Similarly, despite a substantial body of evidence supporting impaired protection via pre- or post-conditioning and GPCR agonists, some report preserved responses to similar stimuli [86, 87]. Reasons for these discrepancies are debated, though disease progression and the presence of dyslipidemia appear to be important. While infarct enlargement is observed across species and models of T1DM and T2DM [2], infarct reduction is predominantly identified in rodent models of acute streptozotocin (STZ) dependent hyperglycemia [2, 86, 88–90]. This may reflect distinct impacts of acute (0–6 week) vs. established or chronic disease. While some also report apparent cardioprotection in models of T2DM [91], this may similarly reflect distinct changes on early transition to T2DM vs. established disease [12, 92]. Presence or absence of dyslipidemia may also be important, with some evidence hypercholesterolemia has opposing effects on infarct tolerance compared with hyperglycemia alone [93]. Mechanisms implicated in differing ischemic tolerance in acute vs. chronic DM include shifts in PI3K/Akt [12, 94, 95] and extracellular signal-regulated kinase 1/2 (ERK1/2) signaling [90], mitochondrial glucose oxidation and malate-aspartate shuttle function [92], and capillary density, vascular endothelial growth factor (VEGF) expression and endothelial nitric oxide synthase (eNOS) signaling [94]. Clinically, the negative impacts of chronic disease are most relevant regarding infarction and cardioprotection, with acute effects relevant only during transition to disease and potentially on cessation of therapy. Almost universally, observations support worsened myocardial ischemic tolerance in models of chronic T1DM or T2DM, with the weight of evidence supporting associated failure in diverse cardioprotective responses.

#### T1DM and infarction

A range of studies report worsened infarction in experimental models of T1DM [96–100] while some report no effect on infarct tolerance [6, 13, 101–112], or protection against both infarction [86, 88, 89, 113] and contractile dysfunction [114]. However, as alluded to above, a biphasic pattern may emerge in STZ-dependent rodent models with evidence of early protection followed by restoration or worsening of infarct tolerance beyond 6–8 weeks. Protection against infarction evident 1–4 weeks after STZ challenge is lost from 8 weeks [115], while reduced ischemic tolerance may emerge by 20 weeks [90] (in association with impaired ERK1/2 phospho-activation). Ma et al. [94] report that protection against infarction and caspase-3 activation in T1DM rats is transient, apparent at 2 weeks and lost by 6 weeks, in association with transient changes in capillary density, VEGF expression, Akt phosphorylation, and eNOS expression. Similarly, early protection against arrhythmogenesis at 2 weeks (with improved maintenance of Na^+^, Ca^2+^, K^+^ and Mg^2+^) transitions to worsened outcomes after 8 weeks in T1DM rats [116]. Acute hyperglycemia itself has been shown to worsen myocardial infarction [96, 102, 117–124], exert no effect [125–128], or less commonly to reduce infarction [129]. Reasons for these disparate observations are unclear, and together with the basis of apparently opposing effects of early vs. late hyperglycemia in rat models of T1DM, warrant further investigation.

#### T1DM and cardioprotection

Beyond a transient intrinsic protection in the early stages of STZ-induced hyperglycemia [2, 86, 88–90], studies report inhibition or complete loss of cardioprotective responses in rodent models of T1DM [91, 130–132]. Protective ‘conditioning’ responses negated or inhibited include ischemic pre- [110, 116, 133] and post-conditioning [102, 104, 106, 108, 134, 135], delayed protection with ischemic preconditioning [102], hyperoxic preconditioning [113], and remote post-conditioning [109]. Protective responses to pharmacological stimuli including anesthetic post-conditioning [101, 104, 111], ACE inhibition [108], opioid [103, 107, 112] and adenosine GPCR agonism [100], and adiponectin [135] and cytokine [13] receptor activation are also lost in T1DM. Przyklenk and colleagues [134] present evidence post-conditioning may actually exaggerate injury in the context of T1DM. Acute hyperglycemia also inhibits cardioprotective responses, blocking ischemic pre- [102, 120] and post-conditioning [121], remote ischemic perconditioning [127], anesthetic pre- [119] and post-conditioning [125, 128], together with glucose-insulin-potassium (GIK) protection [118]. Nonetheless, there are some reports of preserved protection in models of T1DM, including exercise [136] and ischemic preconditioning [86], while Potier et al. [108] identify a specific shift to protective efficacy of B2 bradykinin receptors in T2DM hearts (vs. B1 receptors in non-DM tissue). Atorvastatin is also reportedly cardioprotective in T1DM rats [137], involving a glycogen synthase kinase 3β (GSK3β) dependent protection linked to heat shock factor 1 and heat shock protein 70 (HSP70).

#### T2DM and infarction

Elements of T2DM individually modify infarct tolerance and cardioprotective signaling, including dyslipidemia [138–140], insulin-resistance and hyperglycemia [102, 118, 120–122]. Studies identify exaggerated infarction and contractile dysfunction in different models of T2DM [132, 141–146]. Nonetheless, there are also reports of unchanged infarct tolerance [87, 126, 134, 147–150] or reduced infarction in models of T2DM. The latter reductions are observed 5 days post STZ injection in high-fat fed rats (a protection negated by hypercholesterolemia) [93], and at 16 weeks in Zucker diabetic fatty (ZDF) and lean Goto-Kakizaki (GK) T2DM rats [91, 94]. As for T1DM, disease progression appears to be key, with evidence of a transient protection during disease onset that is lost with T2DM progression in GK rats [90], while infarct-intolerance also emerges with chronic T2DM in ZDF rats [92]. These latter studies link the evolution of infarct tolerance with established T2DM to shifts in Akt signaling, suppression of malate-aspartate shuttle proteins and impaired post-ischemic recovery of glucose oxidation.

#### T2DM and cardioprotection

Diverse cardioprotective responses are impaired or negated in models of T2DM, including loss of ischemic pre- [141] and post-conditioning [134, 142, 145], He-induced pre- and post-conditioning [147], and protection via anesthetic [49], erythropoietin [13, 132], adiponectin [151], and β_3_-adrenergic receptor activation [146]. Interestingly, and consistent with membrane dysfunction, T2DM abolishes the cardioprotective potential of human and rat exosomes [15]. Exosomes are ~ 100 nm lipid bilayer vesicle derivatives of endosomes that play a role in transmitting protective signals between cells and tissues [152]. Cardioprotection may involve exosomal HSP70-dependent activation of myocyte toll like receptor 4 and reperfusion injury salvage kinase (RISK) signaling [153]. Failure of exosomes to induce protection in DM appears to involve abnormal vesicle structure/function rather than impaired protective signaling since exosomes from healthy donors are protective [15]. While exosome size was unaltered, contents of CD81 and HSP70 were increased in DM. Given evidence of exosome involvement in endogenous protection, this dysfunction may contribute to impairment of both conditioning responses and intrinsic ischemic tolerance.

Conversely, there are reports of preserved cardioprotective responses in T2DM, including efficacy of far red/near infrared light [126], sphingosine-1-phosphate [87], peroxisome proliferator-activated receptor γ (PPARγ) activation [150], post-ischemic glutamate [154] and H_2_S preconditioning [155]. Exercise may also retain efficacy, improving ischemic tolerance in the hearts of T2DM (GK) rats [146, 156], and glycemic state and ischemic tolerance in obese mice subjected to 20 weeks of high-fat feeding [157]. Those cardioprotective modalities consistently preserved in different models of DM demand further focused study as potentially efficacious therapeutic candidates.

### Summary

While somewhat contentious, studies of human and animal myocardium generally support detrimental effects of both T1 and T2DM on myocardial ischemic tolerance and cardioprotection (Table 1). Mechanistic interrogation supports a complex pathogenesis, including signaling dysfunction (e.g. impaired PI3K/Akt signaling) [5, 12–14, 60, 90, 94], and abnormalities in mitochondrial function and quality control [10, 19, 20], ubiquitin–proteasome system activity [16], oxidant/anti-oxidant systems [10, 11], and gene and miRNA expression [18]. Influencing many of these potential effector mechanisms, the sarcolemma plays an overarching role in governing ischemic tolerance and cardioprotection. Cardiac sarcolemmal changes arise in DM (see Table 2), reflecting altered lipid metabolism and incorporation, modification of resident lipids and proteins, and significant structural and functional remodeling of caveolae [158, 159], T-tubules [160, 161] and gap junctions [162]. Detailed further below, such changes modify the fundamental biophysical properties of the membrane, glucose and fatty acid utilization, ion channel function, propensity to membrane disruption, and signaling via the insulin receptor (InsR) and receptors governing cardiac stress, growth and death responses.

**Table 1:** Changes in myocardial ischemic tolerance and cardioprotection in animal models of DM

Species—model	Duration or age	Ischemic tolerance	Effect on cardioprotection	Ref.
Type 1 DM				
Mouse—STZ	1 week	⇓	⇓ RPostC	[109]
	2 week	⇔	⇓ IPostC	[134]
	4–5 week	⇔	⇓ IPostC, ⇓ ACE inhibition	[108]
Rat—STZ	1 week	⇑	⇓ HOPreC	[113]
	2 week	⇑	⇔ IPreC	[116]
	2 week	⇔	⇓ Opioid	[107]
	2 week	⇔	⇓ Opioid	[112]
	2 week	⇔	⇓ Opioid	[298]
	2 week	⇓	⇓ Sevoflurane	[106]
	4 week	⇔	⇓ Erythropoietin	[13]
	4 week	⇓	⇓ APN, ⇓ IPostC	[135]
	4–5 week	⇓	⇓ IPostC, ⇓ Sevoflurane	[104]
	6 week	⇓	⇓ IPreC	[133]
	6 week	⇔	⇓ IPreC	[110]
	8 week	⇓	⇓ IPostC	[99]
	8 week	⇓	⇓ APN, ⇓ IPostC	[135]
	8 week	⇓	⇓ IPreC	[116]
	8 week	⇓	⇓ Adenosine	[90]
	9 week	⇔	⇓ Sevoflurane	[111]
	12 week	⇓	⇓ IPostC	[121]
	Unreported	⇔	⇓ Opioid	[103]
Dog—alloxan/STZ	3 week	⇔	⇓ Isoflurane	[101]
	3 week	⇔	⇓ IPreC	[130]
Rabbit—alloxan	5–6 week	⇔	⇓ LPreC	[102]
TYPE 2 DM				
Mouse—HFD	8 week	⇓	⇓APN	[151]
	12 week	⇓	⇓β_3_-AR	[146]
Mouse—*ob/ob*	8–10 week old	⇓	⇓IPreC	[142]
Mouse—*db/db*	10–12 week old	⇓	⇓IPostC	[145]
	12–14 week old	⇔	⇓IPostC	[134]
	Unreported	⇔	⇔Infra-red light	[126]
	12 week old	Not tested	⇔H_2_S PreC	[155]
Rat—STZ/HFD	6 week	⇔	⇔S1P	[87]
Rat—HFD	4 week	⇔	⇔Erythropoietin	[13]
	8 week	⇑	⇓Sevoflurane	[496]
Rat—ZDF	12 week old	⇔	⇔Glutamate	[154]
	16 week old	⇑	⇓IPreC	[91]
Rat—ZO	10–12 week old	⇓	⇓IPreC, ⇓Diazoxide, ⇓HePreC	[141]
Rat—GK	12 week old	⇔	⇓PPAR	[150]
Rat—OLETF	25–30 week old	⇓	⇓Erythropoietin	[132]
Rat—mtFHH	12–14 week old	⇔	⇓Isoflurane	[149]

*HFD* high fat diet, *ZDF* Zucker diabetic fatty, *ZO* Zucker obese, *GK* Goto-Kakizaki, *OLETF* Otsuka Long-Evans-Tokushima fatty, *mtFHH* T2D crossbreed with mtDNA from fawn hooded hypertensive rats, *IPreC* ischaemic preconditioning, *IPostC* ischaemic postconditioning, *HOPreC* hyperoxic preconditioning, *HePreC* helium preconditioning, *LPreC* ischaemic late preconditioning, *RPreC* remote preconditioning, *S1P* sphingosine-1-phosphate, *APN* adiponectin, *β*_*3*_ *-AR* β_3_-adrenergic receptor, *w* weeks

**Table 2:** Cardiac sarcolemmal composition changes in models of T1DM

Sample	Chol	FFA	TRI	Phospholipid	Saturated FAs	Unsaturated FAs	Ref.
Heart			⇑	⇔ ∑PL			[191]
Ventricle		⇑	⇑				[192]
Ventricle				⇑ LPC ⇓ PE, CL ⇔ PI, PS			[196]
Heart						⇓ 20:4, 22:4, 22:5 ⇑ 18:2, 20:3, 20:5	[197]
Heart			⇑	⇔ PC ⇔ PE	(PC) ⇓16:0 ⇑18:0	(PC) ⇓ 20:4, ⇑ 18:2 (PE) ⇓ 20:4	[193]
Heart					(PE) ⇓ 18:0 (PC) ⇓ 16:0	⇓ 22:4 (PE)	[201]
Heart				⇔ CGP ⇔ EGP	(CGP) ⇓ 16:0	(CGP) ⇓ 20:4, ⇑ 18:2 (EGP) ⇑ 18:2	[200]
Ventricle				⇑ EGP	(CGP, EGP) ⇓ 16:0 (EGP) ⇓ 18:0	(CGP, EGP) ⇓ 22:6, 20:4 ⇑ 18:2	[360]
Heart				⇑ EGP, PME, PI	(EGP) ⇑ 18:0, 16:0 (TRI, NEFA) ⇑ 16:0	(EGP) ⇑ 18:2 (TRI) ⇑ 18:1, ⇓ 18:2	[202]
Sarcol-emma	⇑			⇑ ∑PL, CGP, EGP, SGP ⇓ SM	(PC) ⇑ 16:0 (PMC) ⇑ 18:0 (PE) ⇓ 16:0 (PME) ⇓ 18:0, ⇑ 16:0 (PS) ⇑ 16:0, ⇓ 18:0	(PC) ⇓ 20:4, ⇑ 18:2, 18:3 (PMC) ⇑ 18:2, ⇓ 20:4 (PE) ⇓ 20:4 (PME) ⇑ 18:2, ⇓ 20:4 (PS) ⇓ 22:6, 20:4, ⇑ C18:1	[199]
Heart	⇑	⇑	⇑	⇑ ∑PL			[194]
Heart	⇑	⇑	⇑	⇑ PE, SM, LPL ⇓ PC, PI + PS	⇑ ∑Sat FA	⇓ ∑Unsat FA, ⇓ ∑n − 3, ⇓ ∑n − 6	[195]

Changes (up or down) in levels of myocardial or sarcolemmal lipids in models of T1DM are summarized. Sarcolemmal lipid changes are not well defined in models of T2DM. Changes in specific saturated and unsaturated fatty acids species are indicated, with shortened numerical descriptions reflecting numbers of carbons and double bonds (e.g. palmitic acid, 16:0; stearic acid, 18:0; linoleic acid, 18:2; docosahexaenoic acid, 22:6)

*CGP* choline glycerophospholipids, *EGP* ethanolamine glycerophospholipids, *IGP* inositol glycerophospholipids, *Chol* cholesterol, *CL* cardiolipin, *FAs* fatty acids, *FFA* free fatty acid, *LPC* lysophosphatidylcholine, *LPL* lysophospholipid, *NEFA* non-esterified fatty acid, *PC* phosphatidylcholine, *PE* phosphatidylethanolamine, *PI* phosphatidylinositol, *PL* phospholipid, *PMC* plasmenylcholine, *PME* plasmenylethanolamine, *PS* phosphatidylserine, *SGP* serine glycerophospholipids, *SM* sphingomyelin, *TRI* triglyceride

## Sarcolemmal changes in DM

Though research has largely focused on intracellular and metabolic determinants of cardiac stress responses in DM, the sarcolemma plays a key role in governing these and other changes and warrants further research attention [21]. The sarcolemma represents the myocytes structural bounds, and is the primary environmental and inter-cellular interface; a scaffold for ion channel, receptor, transport and mechano-transduction complexes, and medium for detection of intra- and extra-cellular stressors. It is thus intimately involved in receptor signaling, ion homeostasis, substrate delivery, inflammatory and immune function, and detection and transduction of physico-chemical changes. As the site of glucose and fatty acid uptake and InsR signaling, the sarcolemma and its microdomains are a fundamental substrate for the metabolic dysregulation in DM. Molecular modification and disruption of the sarcolemma can thus contribute to multiple aspects of myocardial dysfunction and pathology in DM.

**Fig. 1:**
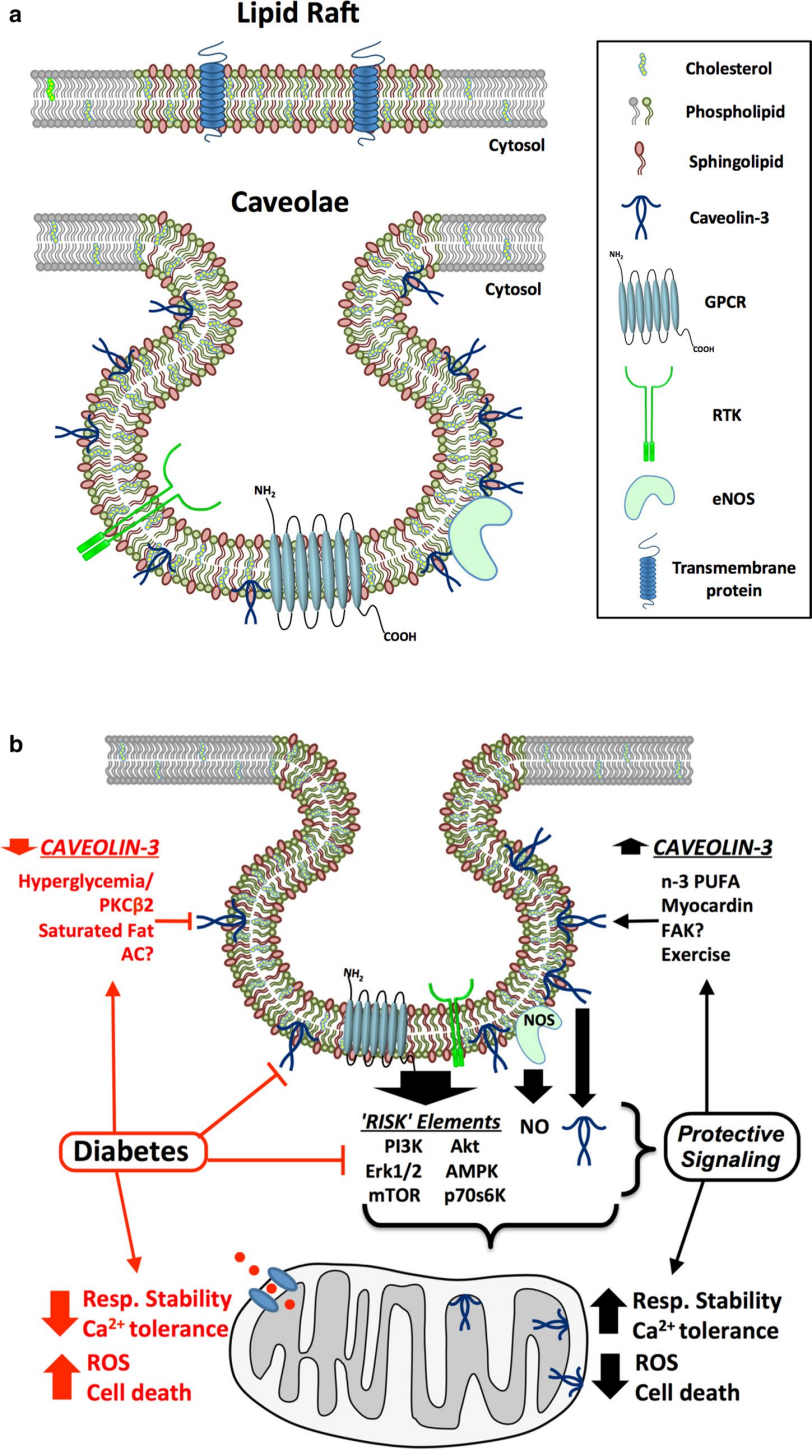
**a** Sarcolemmal makeup and caveolar domains. Planar lipid rafts are more ordered elements of the sarcolemma, containing greater sphingolipid and cholesterol levels and forming signaling microdomain platforms. A subset of rafts, caveolae, localize signaling integral to ischemic tolerance and cardioprotection, including NOS, GPCRs, RTKs and coupled effector molecules. Caveolins are critical to caveolae formation and function and protective signaling. **b** Modulation of caveolae/caveolins and related cardioprotective signaling in DM. Diabetes may exaggerate mitochondrial dysfunction and associated death, while individual elements of DM may disrupt caveolar control and caveolin expression: (i) hyperglycemia-dependent PKCβ2 activation may suppress caveolin-3 expression/localization; (ii) saturated fats (e.g. palmitate) may displace or depress caveolin-3. Disruption of caveolar control and caveolins will limit protective signaling to mitochondria, including caveolin-3 translocation/modulation. Potential determinants of caveolin-3 expression and caveolar function include PKCβ2, saturated fats vs. n-3 PUFAs, AC (adenylate cyclase) and FAK (focal adhesion kinase) signaling, myocardin activity and physical activity

Structurally the sarcolemma is a dynamic fluid bilayer of phospholipids, comprising complex assemblies of proteins, cholesterol and other lipids (Fig. 1a). Within this lipid sea float organized clusters of sphingolipids and cholesterol that form distinct microdomains known as lipid rafts. An important sub-set of these rafts, the caveolae are small invaginations (50–100 nm in diameter) that appear particularly relevant in DM and its cardiac sequelae [158, 159, 163]. Among other functions these ‘little caves’ serve as structural and regulatory platforms for receptor, ion channel and transporter proteins [164–166]; participate in mechanotransduction, protection against disruption and regulation of membrane repair [167]; and govern cardioprotective signaling [168–170]. Lipid rafts can also serve as redox signaling platforms that recruit and assemble nicotinamide adenine dinucleotide (NADPH) oxidase subunits and related proteins [171, 172]. The functional properties of the sarcolemma and its microdomains are governed by molecular composition, which is sensitive to diet, physical activity, genetic makeup and disease, and is significantly disturbed in DM (Fig. 1b, Table 2).

### Changes in sarcolemmal lipid profiles and function in DM

The biophysical properties of the membrane determine resident protein conformation, mobility and function. Fluidity or viscosity governs molecular motion and interactions within this lipid bilayer, thereby influencing the functionality of receptors, transporters and ion channels [173]. Fluidity is determined by lipid makeup, including: the tightness of acyl-chain assembly on phospholipid molecules; degree of phospholipid saturation; and local ratios of cholesterol, lipids and proteins. Membranes rich in cholesterol and tightly packed acyl chains possess greater rigidity, impacting movement and interaction of receptors and other molecules. Changes in sarcolemmal biophysics as a result of altered lipid metabolism do appear causally important in DM [174]: InsR signaling is inhibited by reductions in membrane fluidity, and both glucose transporter type 4 (GLUT-4) transport to the membrane [21, 174] and glucose uptake [175–177] parallel membrane fluidity changes. Analysis of hepatic cells supports causal involvement of sphingomyelin-dependent lipid microdomain changes in insulin-resistance and T2DM [178]. Inflammation, important in DM and cardiovascular dysfunction, is also promoted by abnormalities in membrane phospholipid and polyunsaturated fatty acid (PUFA) composition [179–181]. Positive feedbacks arise, whereby effects of DM and dyslipidemia on sarcolemmal signaling evoke further lipid accumulation and membrane dysfunction. For example, up-regulation of G protein-coupled receptor kinase 2 (GRK-2) with DM or high-fat feeding inhibits GPCR and InsR signaling, promoting further lipid accumulation, insulin-resistance and hypertrophy [182], with recent evidence these changes in GRK-2 also inhibits cardioprotective opioid receptor signaling [183]. The fundamental biophysical properties of the membrane can thus strongly influence the development and pathological impacts of DM across cell types.

Initial studies in DM identified reduced erythrocytes membrane fluidity [184, 185], a change evident even in newly formed cells [186] and subsequently confirmed via different approaches in multiple cell types [21, 187, 188], including cardiomyocytes [189]. Reduced fluidity is broadly consistent with increased membrane content of lipids promoting rigidity, including cholesterol, sphingomyelin and saturated fatty acids [187, 190] (Table 2). It is nonetheless interesting to note that erythrocytes normally possess relatively little cholesterol and lack caveolins. Specific myocardial analyses support increased levels of cholesterol and fatty acid saturation vs. desaturation and differential changes in phospholipids and PUFAs, though studies are limited to models of T1DM/hyperglycemia.

In early work, Denton and Randle [191] found a twofold increase in myocardial glycerides (predominantly triglycerides) in alloxan-induced T1DM in rats without significant changes in phospholipid content, though sarcolemmal fractions were not specifically examined. Increased cardiac triglyceride were confirmed in T1DM rat hearts, together with elevations in free fatty acid levels [192–195]. Early study of phospholipid content revealed reductions in sarcolemmal cardiolipin and phosphatidylethanolamine (PE) vs. elevated lysophosphatidylcholine levels in T1DM hearts, while phosphatidylinositol (PI) and phosphatidylserine (PS) were unchanged [196]. Later analysis of phospholipid makeup in hearts from T1DM rats revealed phospholipid depletion of n-6 arachidonic acid (AA; C20:4), docosatetraenoic acid (C22:4) and docosapentaenoic acid (C22:5) species, whereas contents of n-6 linoleic (C18:2) and dihomo-γ-linolenic acids (C20:3) and n-3 eicosapentaenoic acid (C20:5) were increased [197]. Subsequent studies confirm phospholipid depletion of AA and also palmitic acid (16:0) vs. enrichment with linoleic and dihomo-γ-linolenic acids [193, 198–200] (Table 2), though Black et al. [201] found no change in phospholipid AA content in T1DM rat hearts (while phospholipid stearic acid and palmitate levels fell). Han et al. [202] reported three major sarcolemmal changes in T1DM rats: a reduced ratio of saturated:unsaturated PE species; increased PI and plasmenylethanolamine; and remodeling of triacylglycerol species. More recent analysis in alloxan-induced T1DM in rats confirms increased cardiac cholesterol, free fatty acids, triglycerides and lipid saturation, reduced de-saturation and n-3 and n-6 PUFA levels, and differential changes in phospholipids including increased PE, sphingomyelin and lysophospholipid vs. reduced phosphatidylcholine (PC) and PI + PS [194, 195]. Collectively, these studies support cholesterol, triglyceride and free fatty acid accumulation, increased saturation vs. desaturation, and remodeling of the major choline and ethanolamine phospholipids, with loss of AA and accumulation of linoleic acid and dihomo-γ-linolenic species (Table 2).

Shifts in cholesterol and fatty acid saturation are important to changes in membrane biophysics. Membrane fluidity is particularly dependent upon cholesterol content, which is consistently increased in models of T1DM [194, 195], in association with reduced fluidity and Ca^2+^ influx [173, 203]. Indeed, membrane cholesterol changes are likely to contribute in multiple cardiovascular disorders [204]. Cholesterol molecules provide structural support, function as molecular ‘glue’ for lipid raft assembly, promote curvature of the membrane [205], and are important to caveolae formation [206]. Control of cholesterol is therefore essential to maintenance of membrane architecture, fluidity and microdomain formation. Shifts in the degree of fatty acid saturation also influence fluidity, with DM consistently increasing cardiac fatty acid levels and saturation vs. desaturation [193, 198]. Changes in phospholipid profiles additionally alter fundamental biophysical properties [207], together with protein activity [208, 209], recruitment of signal proteins [210], propensity for fusion [211] and production of lipid second messengers [209, 212]. Sarcolemmal phospholipids may also undergo relevant post-translation modification in DM, for example altered *N*-methylation [213] may alter cardiac sarcolemmal Ca^2+^ fluxes [214, 215].

Polyunsaturated fatty acids exert complex effects on membrane structure and function [216]. For example, n-3 PUFAs such as eicosapentaenoic (EPA) and docosahexaenoic acid (DHA) remodel cholesterol-enriched lipid microdomains, with evidence their incorporation increases molecular order (despite their high disorder). Differences in lipid microdomain interactions of EPA and DHA may lead to differential changes in ‘bioactivity’ [216]. Both in vitro [217, 218] and in vivo studies [219, 220] indicate n-3 PUFAs incorporate into membrane fractions corresponding to rafts, generally within the most abundant and DM-sensitive phospholipids (PE and PC), and magnetic resonance studies show differential changes in membrane structure when n-3 PUFAs are incorporated into PE vs. PC, the phospholipid fractions predominantly modified in DM [193, 195, 198, 199]. Lipid raft incorporation of n-3 PUFAs is accompanied by reduced levels of the highly disordered n-6 PUFA AA [179], consistent with declining sarcolemmal AA species in DM [193, 198, 199]. Several studies show n-3 PUFAs can lower raft cholesterol levels, which may underlie effects on protein lateral organization and signaling [221]. Impacts on cholesterol may include shunting from raft to detergent-soluble membrane fractions in some cell types [221, 222], potentially reflecting the poor affinity of n-3 PUFAs for cholesterol [223]. However, in some cell types reductions in raft cholesterol are not matched by changes in the detergent-soluble fraction [224], which may be related to the ability of n-3 PUFA to promote internalization of lipid microdomains (including raft cholesterol) [225]. An area of interest has been the influence of PUFAs on ion channel function, electrical stability and arrhythmogenesis, though mechanisms underlying such effects are yet to be fully detailed [226, 227].

Membrane dynamics are also influenced by glycation and associated free radical production [173, 203]. Treatment with the anti-glycation and anti-oxidant compound resorcylidene aminoguanidine (RAG) reverses DM-dependent reductions in cell membrane fluidity [173]. Profoundly reduced sensitivity to Ca^2+^ overload in myocardium from DM rats is also inhibited [173], additionally highlighting the importance of fluidity to ion homeostasis in DM hearts. Roles of such post-translational changes are discussed in more detail further below.

Unfortunately, studies of sarcolemmal changes in DM have focused to date on models of T1DM, with little to no information regarding changes in T2DM (Table 2). Moreover, investigations have yet to detail temporal patterns of sarcolemmal change during DM development and progression. It would be of great value to undertake such time-course analyses across what appears the critical range for variable shifts in ischemic tolerance (i.e. no effects or improved tolerance from weeks 1–6; reduced tolerance at later times), permitting correlation of membrane makeup and ischemic tolerance changes. Membrane lipid analyses have been undertaken within yet not across these differing time periods (Table 2), with broadly similar lipid changes reported at 2–6 weeks [195, 201, 202] and 8–9 weeks [197, 199, 200]. No distinguishing feature is evident in later membrane profiles, though there is only a single study at ≥ 12 weeks [193]. There is also no information on the time-course of changes in caveolae and caveolin proteins, another knowledge gap deserving attention.

### Remodeling of sarcolemmal microdomains

Membrane proteins and signaling are compartmentalized between specialized microdomains rich in cholesterol and sphingolipids vs. other membrane regions [228]. Though a simplified model given the true complexity of the plasma membrane [229], sarcolemmal assemblies of lipids and proteins may be divided into either lipid rafts or planar platforms (loosely corresponding to detergent insoluble membrane fractions) and non-raft domains (corresponding to detergent soluble membrane). These domains differ markedly in their ion channel, transporter, receptor and signaling protein profiles [230–232]. Altered membrane composition can thus disrupt signaling, ion movement and substrate transport through differential changes in lipid raft/caveolar vs. non-raft domains. However, the compartmentation of proteins between raft and non-raft regions remains a controversial topic, reflecting in part varying outcomes with different membrane fractionation methodologies.

#### Lipid raft vs. non-raft proteins

These distinct regions may be differentially modified in DM, although studies of the cardiac sarcolemma are limited. For example T2DM *db/db* mice exhibit ~ tenfold elevations in the area of raft clusters in aortic endothelium, confirming that increased cellular lipid content can drive raft cluster formation [233]. Both horizontal and vertical clustering of rafts is observed, increasing the height of these aggregates [233]. Such changes will modify resident protein integration and function. Sequestration of specific ion channel, receptor and transporter proteins within raft microdomains is an important means of compartmentalizing and specifying downstream signal transduction by the sarcolemma. For example, caveolar localization ensures signaling specificity of cardiomyocyte β-adrenoceptors, limiting non-selective effects on sarcoplasmic reticulum (SR) and myofilament function [234]. A sub-population of L-type Ca^2+^ channels (LTCCs) has also been identified in caveolae domains that appears critical in regulating β-adrenoceptor [235] and hypertrophic calcineurin/nuclear factor of activated T-cells (NFAT) signaling [236]. Evidence also supports functional localization of LTCCs to caveolae in human and rodent atrial myocytes, with a caveolae-targeted LTCC antagonist inhibiting Ca^2+^ fluxes [237]. In contrast, a recent report concludes that neither a caveolae targeted LTCC activator or inhibitor modifies function or hypertrophic responses in murine hearts [238].

Regulation of other important channels and pumps may be dependent upon caveolae domains. For example, despite evidence cardiac Na^+^-K^+^-ATPase localizes to non-raft regions [239], there is also evidence for caveolae/caveolin association and control [240]. Almost half of the Na^+^-K^+^-ATPase α1-subunit and nearly all of the glycosylated β1-subunit reportedly localizes to cardiac caveolae [241], and caveolin-1 deletion inhibits interactions between Na^+^-K^+^-ATPase, caveolin-3 and PI3K in cardiomyocytes [242]. Thus, inhibition of sarcolemmal Na^+^-K^+^-ATPase sub-unit expression and activity in STZ-dependent T1DM models [243, 244] may reflect a caveolae specific response. Such an effect is consistent with predicted outcomes of cholesterol accumulation [245]. Indeed, high dietary cholesterol also reduces Na^+^-K^+^-ATPase [246], and cholesterol may render sarcolemmal penetration of the ATPase complex energetically unfavorable, while reducing surface charge density is chemically unfavorable [245]. Similarly, the cardiac Na^+^/Ca^2+^ exchanger is suppressed in DM [247], and despite some evidence it does not localize to rafts or caveolae [239], there is also evidence the exchanger interacts with caveolin-3 in sarcolemmal vesicles [248], and its activity is depressed with cholesterol depletion [249]. Further work may clarify the impacts of DM on cardiac raft and non-raft ion channels and pumps.

#### Caveolar membrane microdomains

Evidence accumulated over the past decade highlights a particular importance of caveolae in protection of myocardium against metabolic (ischemia, hypoxia) and mechanical stressors [167–170], together with perturbations and potential involvement in cardiac disease [159, 163]. Few studies have examined effects of DM on caveolar structure and density, although constituent caveolin proteins are significantly modified [135, 250–253] and caveolar localization of signaling molecules altered [250, 251, 254]. Evidence implicates abnormal caveolar control in the development and cardiac-specific effects of DM [158, 159].

Caveolae have at least four major functions: (i) as signaling platforms in the membrane, for example for receptor tyrosine kinases (RTKs) including the InsR [131], GPCRs [165], eNOS [255], other signaling proteins [256] and ion channels [166]; (ii) regulating fatty acid transport [257, 258] and glucose handling [158]; (iii) participating in mechanotransduction and acting as membrane ‘reservoirs’ to limit damage with mechanical stress [167, 259]; and (iv) functioning as membrane transport vesicles, budding from the membrane in response to specific cues and participating in membrane repair [260]. Abnormalities within these regulatory domains will thus influence ion and substrate movement, protective signaling and myocyte responses to mechanical perturbation, impairing cardiac responses to both pathologic insult and potential therapies.

As for lipid rafts in general, caveolae formation and function are dependent upon lipid composition, particularly cholesterol and sphingolipid content [228, 261, 262]. A key distinguishing feature is the presence of cholesterol-associated caveolin proteins, involved in stabilizing the physical architecture of these flasks and regulating signaling and transport processes [228]. Depletion of membrane cholesterol [263] or caveolins [264] inhibits caveolae formation and negates myocardial responses to diverse protective stimuli [265, 266]. Cholesterol depletion also disrupts the Z-band localization of caveolin-3 in cardiomyocytes, and alters cytoskeletal architecture [267]. Highly abundant PS and phosphatidylinositol (4,5)-bisphosphate [PIP2] may also be important, concentrating within caveolae and functionally compartmentalizing lipid pools [268–270]. Caveolae depletion with caveolin-1 knockout or depletion leads to re-organization of plasma membrane PS domains [271], and consistently down-regulates pathways of lipid metabolism across cells [272]. There is evidence the caveolin scaffolding domain—a 20-amino acid sequence initially implicated in controlling signal molecules—has an intrinsic capacity to concentrate local cholesterol, PS and PIP2 [273]. Effectively enriching caveolar oligomers in PS and PIP2, this process is proposed as a means of attracting membrane-sensing cavin proteins to initiate a cascade of further caveolin, PIP2 and PS recruitment to membrane rafts [274]. Cholesterol concentration around caveolin oligomers may modify biophysical properties to favor membrane bending by the cavin-caveolin coat complex. These inter-dependencies provide a basis for the sensitivity of caveolae formation and function to cholesterol and phospholipids, the levels of which are perturbed in DM [195, 196, 199–202]. Given evidence of cholesterol accumulation in the sarcolemma of DM hearts, a scenario of both caveolar disruption via caveolin suppression and reduced membrane fluidity via cholesterol accumulation could arise. How accumulated cholesterol is distributed between microdomains in DM is not clear, however it is possible fluidity within depleted caveolae populations is compromised.

#### Caveolar “coat proteins”—the caveolins and cavin families

While there are few analyses of cardiac caveolar architecture and density in DM, significant changes in constituent caveolins and cavins are observed and likely disrupt caveolae function and formation. Hyperglycemia may suppress myocardial caveolin-3 in a PKCβ2 dependent manner [250] and H9c2 cardiomyoblast caveolin-3 in an oxidant-dependent manner [275], while hyperinsulinemia also depresses caveolar caveolin-3 in H9c2 cells [254]. Moreover, saturated fats reduce cardiac caveolin-3 [276], as does aging [277], whereas PUFA supplementation can up-regulate caveolin-3 expression [278]. Caveolin-1, in contrast, may be significantly up-regulated in DM [252, 279].

The caveolin proteins are primary structural and regulatory elements of caveolae [168], though also play important non-caveolar roles [259]. For example, sequestration of active caspase-3 by extra-caveolar caveolins may underlie protective effects of β-receptor antagonism in DM hearts [253]. Three caveolin isoforms have been identified with differing functions and tissue distributions [158, 164, 206, 264]. All are expressed in the central nervous system, with ubiquitous caveolin-1 most highly expressed in endothelium, fibroblasts and pneumocytes, where it appears structurally supported by caveolin-2 hetero-oligomerization. In contrast, caveolin-3 is highly specific to striated muscle and plays crucial roles in cardiac stress sensing/responses and cardioprotection [167–170, 256, 259, 264]. Caveolins preferentially arrange in homo-oligomers of 2 to ~ 16 monomers, forming caveolar assembly units, and may require cholesterol for effective insertion into the membrane [264]. A common feature of all isoforms are scaffold domains where signal molecules including G proteins, PKC and eNOS are proposed to physically interact. However, the basis of regulatory molecular interactions with caveolins remains to be defined [280]. Noted above, these domains also appear important in locally concentrating cholesterol and phospholipids [273]. Caveolin-3 is not only essential to myocardial caveolae formation but is particularly important to stress tolerance and cardioprotection. Myocardial [266] and mitochondrial [281] stress responses are strongly caveolin-3 dependent, as is cardiac protection via ischemic and anesthetic preconditioning [169, 265] and opioid GPCRs [266]. Caveolin-3 also influences cholesterol transport [282], ion handling [235, 283, 284], GLUT4 and glucose metabolism [252, 285, 286], and hypertrophic remodeling [287, 288].

Despite these key roles, the control of myocardial caveolin expression remains to be detailed, though studies in other cells support transcriptional regulation by myocardin. A member of a family of transcriptional co-activators responsive to stress, myocardin up-regulates caveolins and caveolae in smooth muscle cells [289]. Specific cardiac studies are lacking, however human expression data support a close association between *Myocd* and *Cav1* gene levels across tissues, including heart [289]. Myocardin control of caveolin-1 and -2 and cavin-2 appears independent of serum response factor whereas control of cavin-1 is dependent on this transcription factor, providing for differential control of cavin-1 vs. caveolins [289]. Myocyte myocardin may be up-regulated by hyperinsulinemia [290], and in other muscle cell types myocardin is up-regulated by oxidative stress sensitive miR-145 [291]. Importantly, emerging evidence reveals new roles for myocardins in glucose and lipid homeostasis (including via caveolins) [292].

The few studies analyzing myocardial caveolins in T1DM have employed relatively acute models (0–6 weeks), and report a hyperglycemic depression of caveolin-3 [135, 250–253] that may contribute to diastolic dysfunction [250], impaired GLUT4 translocation [252] and I–R intolerance [251]. Nonetheless, the acuteness of STZ-induced hyperglycemia and variable ischemic tolerance in these T1DM models raise questions regarding relevance: paradoxical cardioprotection in the initial weeks in rat T1DM models [2, 84, 293] is not relevant to the ischemic intolerance observed in chronic disease and T2DM. Hyperglycemia also acutely depresses caveolin-3 expression in cardiac myoblasts [275], and hyperinsulinemia suppresses caveolar levels of caveolin-3 in H9c2 myoblasts, which may dysregulate Akt-dependent InsR signaling [254]. No study has comprehensively assessed mechanistic involvement of caveolin-3 in the cardiac sequelae of T2DM, with only a single report of an insignificant fall in cardiac *Cav3* mRNA in the non-obese GK rat model [294].

Inhibitory effects of saturated fatty acids [276, 295] and glucose [250] on cardiac caveolin-3 expression and caveolin-dependent eNOS signaling present plausible mechanisms for reduced cardioprotection in DM. Impaired PI3K/Akt/NOS signaling is characteristic in DM myocardium [12, 90, 94, 95, 146, 250], and these signal elements cluster in caveolae [164–166] where eNOS is regulated by caveolin-1 and -3 [250, 275, 296–298], and Akt signaling is promoted by caveolin-3 [250–252, 299]. Studies in rodent models indicate that DM dysregulation of RISK signaling, including PI3K/Akt and glycogen synthase kinase-3β, underlies impaired protection via cytokine receptors [13], GPCRs [14] and progestin and adiponectin receptors [35]; and RISK-dependent pre- and post-conditioning responses are also inhibited in DM [2, 116, 117, 130]. Inhibition of Akt signaling and ischemic tolerance in T1DM has been linked to caveolin-3 depletion [251], as has disruption of adiponectin receptor cardioprotection [135]. Recent work also implicates oxidant-mediated dysregulation of caveolin-3/eNOS signaling in the ischemic intolerance in T1DM hearts [275]. An increase in caveolin-1, reported by Penumathsa et al. [252] in hearts of T1DM rats and Bucci et al. [279] in aortic tissue, may also inhibit protective signaling, suppressing eNOS activity [296–298, 300] and promoting dephosphorylation of sarcolemma-associated Akt [301]. In support of this, Ajmani et al. [300] report that a ‘caveolin inhibitor’ and sodium nitrite both restore preconditioning in T1DM rat hearts, however significant limitations include multiple non-specific biological actions of the inhibitor employed, and failure to measure caveolin-1 expression or establish diabetic inhibition of preconditioning.

Less is known regarding potential roles of more recently identified cavin proteins [206, 302]. These coat proteins homo- and heteroligomerize (independently of membrane and caveolins) to form specific caveolar sub-complexes, and are involved in orchestrating the cell-specific formation, caveolin/cavin incorporation and structural modeling of caveolae [206, 302]. They may also be released intracellularly with different stressors/stimuli to regulate gene expression and non-caveolar processes. Depletion of cavin-1 (with attendant loss of caveolae) results in elevations in circulating triglycerides, glucose intolerance and hyperinsulinemia [303], and inhibits cardiac ischemic tolerance and stretch responses while exaggerating cellular permeability (potentially via NOS overactivity) [304]. Perturbation of the caveolar system via caveolin-1 depletion or knockout also dysregulates cardiac stress responses [305, 306]. Whether these gene deletion effects reflect distinct roles and influences of cavins and caveolins, or highlight the broader importance of caveolae is presently unclear. However, differences do emerge in the cardiac effects of cavin-1 vs. caveolin knockout [304]. Intriguingly, effects of cavin-1 and caveolin-1 knockout suggest the diastolic dysfunction in DM could involve disruption of sarcolemmal caveolae: caveolar depletion in both cavin-1 [304] and caveolin-1 [307] knockout hearts is associated with significant diastolic dysfunction or stiffening. Caveolae provide an effective membrane reserve to accommodate physical deformation or stretch [167], potentially influencing the compliance of cardiac cells. Although diabetic diastolic dysfunction is attributed to fibrosis/hypertrophy [20], sarcolemmal makeup and specifically caveolae and associated signaling may contribute to this dysfunction [304, 307].

### Changes in sarcolemmal caveolae influence substrate handling

#### Glucose transport

Caveolar domains are important in glucose and lipid transport, and InsR receptor signaling [158, 274]. Myocardial glucose transport via GLUT4 is spatially confined to caveolar domains [158, 308], where InsRs are also localized [309]. Cardiac insulin-resistance and impaired GLUT4 expression and transport in T2DM [310] may involve disruption of caveolae and caveolin-3 with DM [135, 250–253] and high-fat feeding [276, 295]. Indeed, Penumathsa et al. [252] report reduced expression and association of GLUT4 and caveolin-3 in lipid-rafts of T1DM rat hearts.

Activation of the InsR normally leads to a cascade of Akt phospho-activation and phosphorylation of the Rab-GTPase activating TBC1D4/AS160 protein, a distal effector maintaining GLUT4 vesicles within an inactive intracellular pool [311]. This initiates pathways mediating docking and diffusion of GLUT4 vesicles at the plasma membrane [311]. This path not only increases GLUT4 exocytosis but can limit endocytosis to re-distribute plasma membrane GLUT4. However, in cardiac [312] and skeletal myocytes [313] insulin does not influence endocytosis. Nonetheless, GLUT4 endocytosis in skeletal myoblasts is sensitive to energy state (inhibited by mitochondrial uncoupling), and both clathrin-dependent and clathrin/caveolae-independent (yet cholesterol-dependent) endocytosis paths are involved [313]. This energy-sensitive endocytosis reveals non-caveolae effects of cholesterol, for example promoting negative membrane curvature [205]. This not only further highlights the importance of membrane cholesterol, but shows that distinct membrane changes may independently modify GLUT4 exocytosis and GLUT4 endocytosis.

Not only is GLUT4 movement influenced by caveolae and caveolins, but signaling via the InsR is strongly dependent upon these raft elements. Yamamoto et al. [314] first demonstrated positive control of InsR signaling via caveolin-1 and -3, including evidence of direct caveolin interaction with the InsR kinase domain to promote insulin receptor substrate 1 phosphorylation. Caveolin-3, caveolin-1 and the InsR all interact in cardiac myoblasts, and caveolin-3 depletion renders myocytes insulin-resistant while caveolin-3 haplo-insufficiency increases susceptibility to fatty acid induced insulin-resistance [286]. Disruption of caveolae or caveolin-3 expression in DM [135, 250–253] is thus predicted to limit cardiac InsR signaling, although a parallel elevation in caveolin-1 as reported in a rat T1DM model [252] may modulate such effects. Supporting the value of targeting caveolins, insulin-resistance in obese and DM mice is reversed by hepatic overexpression of caveolin-3, which substantially enhances InsR signaling [315]. Nonetheless, basal glucose metabolism appears largely unaltered in hearts lacking either caveolin-3 [288] or caveolin-1 [316], and thus also devoid of caveolae, although skeletal muscle insulin-resistance arises in both models [308, 317]. While suggesting distinct caveolin/caveolar control of substrate metabolism in cardiac vs. skeletal muscle, cardiac InsR signaling and insulin-resistance have yet to be detailed in these knockout models. Lifelong absence of both caveolae and caveolins in these models may also limit their relevance to more moderate and progressive changes in DM. Other analyses confirm that reductions in caveolin-3 inhibit insulin-stimulated glucose uptake in cardiac myoblasts and myocytes [286], and that hyperinsulinemia in cardiac myoblasts reduces caveolar levels of caveolin-3 and insulin-dependent phospho-Akt [254]. Insulin-dependent myocardial glucose uptake is thus predicted to be impaired with reductions in caveolin-3 expression in DM hearts, though this has yet to be directly assessed.

Ubiquitously expressed caveolin-1 may additionally modulate InsR signaling in DM, and cardiac expression is reportedly increased in T1DM rat hearts [252]. Caveolin-1 is also induced by micro-RNAs up-regulated in obesity (miR103, miR107), and their overexpression induces insulin-resistance in an entirely caveolin-1 dependent manner [318]. However, changes in caveolin-1 are not universal in obesity, some dietary interventions may also augment caveolin-1 [319], and distinct from caveolin-3, cardiac expression of caveolin-1 appears repressed with medium-chain triglyceride but not palmitate supplementation [276]. Further work is needed to clarify effects of caveolin-3 and -1 on insulin-dependent glucose uptake and metabolism in myocardium and cardiac myocytes, identifying specific roles of the caveolins themselves vs. caveolae as regulatory platforms, and the effects of moderate and acute vs. prolonged changes in expression (modeling changes in DM, and avoiding limitations of lifelong gene deletion).

#### Fatty acid uptake

Fatty acid transport is also compartmentalized within lipid rafts and caveolae [320], with the regulatory InsR [309]. Accumulation of long-chain fatty acid metabolites is important in development of myocardial insulin-resistance [321, 322], with more prolonged changes involved in later development of heart failure. The major cardiac fatty acid transporters CD36 and fatty acid binding protein (FABP) normally relocate to the sarcolemma from intracellular stores in response to insulin or contraction [322, 323]. Active CD36 specifically localizes to lipid rafts and caveolae where fatty acid uptake activity is promoted, while inactive intracellular CD36 is associated with non-raft fractions [320]. Overexpression of CD36 enhances skeletal muscle fatty acid oxidation and decreases plasma lipids [324], while deletion impairs cardiac fatty acid uptake, though this may be metabolically compensated by increased glucose oxidation [325]. Sarcolemmal CD36 not only governs uptake but targets fatty acids to specific metabolic sites including mitochondria [326], and plays roles in promoting 5′-AMP activated protein kinase (AMPK) signaling, regulating Ca^2+^ signaling and levels, and acting as co-receptor for toll-like receptors [327]. Permanent sarcolemmal relocation of transporters in obesity and DM thus greatly promotes cardiac lipid and lipid metabolite accumulation to impair insulin signaling and glucose utilization [322, 323]. As critical sites of control, sarcolemmal CD36 and FABP are important therapeutic targets for countering myocardial insulin-resistance and cardiomyopathy.

Changes to caveolae and caveolin-1 and -3 in DM are predicted to impact CD36-dependent uptake given functionally relevant caveolar localization and caveolin control. Hearts from caveolin-3 haplo-insufficient mice do express less CD36 in line with differing caveolin-3 levels, though a twofold rise in caveolin-1 suggests potentially complicating adaptation [286]. Lipid raft targeting of CD36 may involve interaction with caveolin-1 based on effects in non-muscle cells [328], and cardiac lipids and fatty acid uptake are also reduced with caveolin-1 knockout [316]. Diabetic up-regulation of caveolin-1 [252] could thus promote lipid uptake, though myocardial CD36 and caveolin-1 are not always linked: for example, cardioprotective isoflurane increases caveolin-1 and caveolae [305] while reducing caveolar CD36 levels [329]. Although lifelong absence of caveolin-3 does not reduce cardiac fatty acid uptake [288], a halving of cholesterol levels and a 40–50% increase in triglycerides confirm major perturbations of fatty acid handling. Importantly, and as noted above, this model reflects a complex phenotype encompassing lifelong absence of caveolin-3 and caveolae (thus caveolae-localized transporters), which likely disrupts potential caveolin-1 control.

### Membrane cholesterol—beyond fluidity and caveolar domains

Changes in membrane cholesterol do not only influence membrane fluidity [189], curvature [205], and caveolar endowment [223, 262, 267], but also govern T-tubule system integrity and excitation–contraction (E–C) coupling [330, 331], contractile function [267, 332], glucose and fatty acid transport [320, 331, 333–336], and functionality of membrane ion channels, receptors and transporters [337–339]. These diverse effects of cholesterol on sarcolemmal architecture and the functionality of associated proteins may contribute to impairment of cardioprotection and ischemic tolerance with hypercholesterolemia [138, 140, 340] and are relevant to the DM myocardium.

For example, there is evidence that increased membrane cholesterol is key to impaired GLUT4 traffic in insulin-resistance and T2DM, though studies have focused on skeletal muscle given its contribution to systemic insulin sensitivity and glucose homeostasis: glucose-intolerant animal models and humans accumulate cholesterol in skeletal muscle membranes [331, 335]; high-fat diets also increase skeletal muscle cholesterol [332]; DM also increases cardiac cholesterol levels [195]; cholesterol depletion with methyl-β-cyclodextrin reversibly and dose-dependently increases plasma membrane GLUT4 incorporation in myotubes [334]; and cholesterol depletion improves glucose homeostasis in high-fat fed animals, together with insulin-dependent GLUT4 translocation and glucose uptake in muscle fibers [335]. The cholesterol depleting agent chromium also improves glycemic control in T2DM patients [341], and activates GLUT4 trafficking and insulin-stimulated glucose transport in a cholesterol- and AMPK-dependent manner [342]. This is consistent with evidence AMPK improves insulin-stimulated GLUT4 control by lowering membrane cholesterol [335]. These observations support regulation of insulin-stimulated GLUT4 translocation via tissue cholesterol content, and suggest cholesterol removal may be useful in countering myocyte insulin-resistance, although cardiac studies are lacking.

Additional to indirect influences on protein confirmation and function, cholesterol recognition/interaction amino acid consensus (CRAC) and more recently CARC (similar to CRAC, with an opposite orientation—hence “CARC”) domains have been identified in transmembrane proteins, including receptors regulating cellular stress responses [337, 343]. Sometimes located within the same transmembrane segment, these CRAC and CARC domains can directly interact with cholesterol in the cytoplasmic leaflet of the plasma membrane. Modulating multiple ion channels [284, 338] and receptors [339, 343–345], the cardiac significance of sarcolemmal cholesterol:protein interactions awaits further study, particularly in the context of DM and metabolic syndrome.

### Potential influences of DM on cardiac phospholipid signaling

**Fig. 2:**
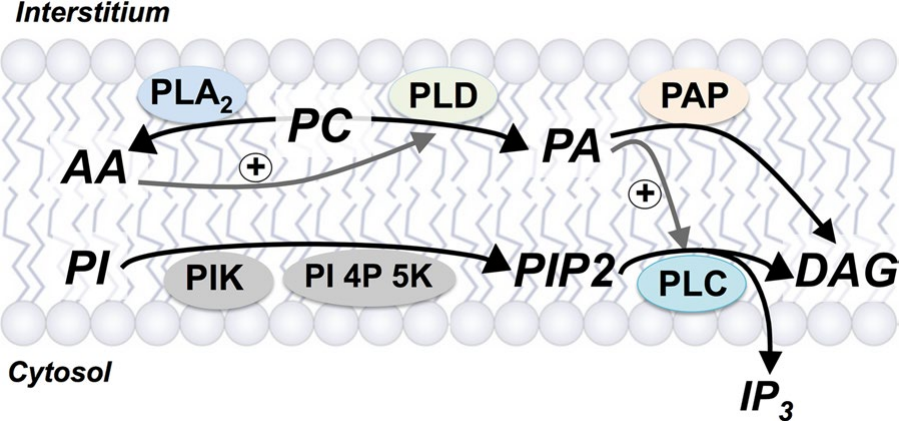
Sarcolemmal phospholipid signaling via phospholipases. *AA* arachidonic acid, *DAG* 1,2-diacylglycerol, *IP3* inositol 1,4,5-triphosphate, *PA* phosphatidic acid, *PC* phosphatidylcholine, *PIP*_*2*_ phosphatidylinositol-4,5-bisphosphate, *PLA*_*2*_ phospholipase A_2_, *PLC* phospholipase C, *PLD* phospholipase D

Membrane lipids not only serve structural roles but are substrates in cell signaling (Fig. 2). Sarcolemmal phospholipids are targeted by three primary phospholipase groups to generate lipid signaling molecules: phospholipases A2, C and D (PLA2, PLC and PLD, respectively). Phospholipid signaling is implicated in cardiac hypertrophy/cardiomyopathy and is perturbed in cardiovascular disease states including DM [346]. Changes observed in sarcolemmal glycerol-phospholipid species in DM rat hearts likely contribute to membrane and contractile dysfunction [195, 199, 202]. In terms of ischemic tolerance, phospholipases are implicated both in mediating and protecting against ischemia–reperfusion injury [347–349]. This may reflect isoform specific effects, including protection via PLCγ_1_ and injury via PLCδ_1_. Shifts in membrane phospholipase signaling may thus contribute to alterations in both infarct tolerance and cardioprotection in DM.

Cardiac PLC activities are reduced in STZ-induced T1DM rats, and basal and phosphatidic acid induced IP3 generation are reduced in cardiomyocytes from DM rats [350]. In contrast, increased PLC activity is implicated in exaggerated α1-adrenergic receptor mediated inotropy with acute (3 day) hyperglycemia [351]. Distinct outcomes with acute vs. chronic DM may be relevant to patterns of early protection and later ischemic intolerance in models of STZ dependent T1DM [2]. A reduced rather than increased PLC activity may contribute to abnormalities in contractility and α1-adrenergic receptor responses with more protracted disease [352]. Reduced PLC generation of 1,2-diacylglycerol (DAG) may impact other cellular processes [353, 354], although myocardial DAG levels are increased with more acute STZ-dependent T1DM and in autoimmune DM (biobreeding) rats [355, 356], which could destabilize the membrane [357, 358].

Phospholipase D activity reportedly declines in DM cardiomyopathy [359], which may limit PA generation and thereby PLC activation. Alterations in AA content of sarcolemmal phospholipids could also reflect dysfunctional PLC signaling [346]. Relatively little is known regarding cardiac PLA2 signaling in DM, however cardiac membrane associated PLA2 activity is increased in rat models of DM [360]. The local environment of caveolae is also important to phospholipid signaling. Up to half of cellular PIP2 is located in caveolin-enriched membrane fractions [268, 361, 362], and this pool is specifically sensitive to GPCR and RTK activation [268] in a cholesterol-dependent manner [263]. For example, the α1 adrenergic receptor (AR) and its Gq effector protein are caveolae localized in adult cardiomyocytes [363, 364], and analysis in neonatal cells possessing both caveolar and non-caveolar PIP2 fractions supports select caveolar depletion upon α1-AR stimulation [365]. Coupled with localized PLC-dependent hydrolysis to DAG, this compartmentation provides for select regulation of caveolar populations of PIP2- and DAG-sensitive ion channels and exchangers [366, 367]. Shifts in caveolar makeup and localized phospholipids thus have capacity to selectively disturb cardiomyocyte receptor signaling and ion channel function.

Critical to cellular growth, substrate metabolism, stress responses and cardioprotection, PI3K isoforms catalyze production of the 3-phosphorylated phosphoinositides phosphatidylinositol 3-phosphate, phosphatidylinositol (3,4)-bisphosphate, and phosphatidylinositol (3,4,5)-triphosphate. While membrane phospholipid pools are modified in DM, it is unclear whether changes are sufficient to influence PI3K signaling. Certainly, dysfunctional PI3K/Akt signaling is implicated in altered InsR control and impaired cardioprotection, among other cardiac changes.

### Membrane glycation, glycosylation, palmitoylation and oxidation in DM

Glycation and enzymatic glycosylation are major factors in the cardiac abnormalities arising in DM [9], as is oxidative stress [10, 11]. Palmitoylation is also an important determinant of sarcolemmal protein function [368–370] and is highly relevant in metabolic disorders such as DM [371, 372], however, diabetic perturbations have been largely studied in non-cardiac tissues. Other modifications may also be relevant in DM, for example reductions in phospholipid *N*-methylation [213] depress Na^+^-dependent Ca^2+^ uptake [214] and enhance ATP-dependent Ca^2+^ efflux [215] in the cardiac sarcolemma of STZ-dependent T1DM models. Such effects may be mechanistically relevant to paradoxical resistance to external Ca^2+^ overload in DM hearts [189].

#### Advanced glycation end-products (AGEs) and the receptor for AGE (RAGE)

Chronic hyperglycemia promotes glycation, the non-enzymatic covalent bonding of carbohydrates to proteins and lipids. Glycation products in turn can form cross-linked structures known as AGEs. These modified proteins/lipids activate cell surface RAGE to trigger ROS generation, activation of nuclear factor kappa-light-chain-enhancer of activated B cells (NFκB) and pro-inflammatory cytokine production. Positive feedback between NFκB and RAGE expression exaggerates ROS and cytokine generation. These processes are implicated in vascular dysfunction in DM, and have been shown to contribute to myocardial changes and dysfunction [373, 374]. Targeting AGE accumulation has also been shown to improve myocardial ischemic tolerance in different models of DM. For example, cardioprotection in rat T1DM models with natural xanthonoid and flavonoids [375, 376] and anti-hyperglycemic glitazones [377] appear to involve inhibition of the AGE-RAGE axis and AGE accumulation. However, cytoplasmic AGE accumulation is typically documented in animal and human tissues, and specific sarcolemmal targets of glycation have not been investigated in detail.

#### Glycosylation

Diabetes increases fluxes through accessory paths of glucose metabolism, including the hexosamine biosynthetic pathway (HBP) that produces the sugar donor for enzyme-mediated β-*O*-linked-*N*-acetylglucosamine (*O*-GlcNAc) modification of proteins or lipids. Studies confirm that increased protein *O*-GlcNAc levels contribute to the cardiac abnormalities of DM. This modulation is complex, however, with *O*-GlcNAc mediating both beneficial and detrimental effects [378]. Transient elevations in *O*-GlcNAc may provide cytoprotection [379], with acutely increased *O*-GlcNAc prior to ischemia or in reperfusion reducing infarction and dysfunction [380]. Inhibition of *O*-linked β-*N*-acetylglucosamine transferase (OGT) can also inhibit cardioprotection [381] while inhibition of protein *O*-GlcNAcase (OGA) may improve cardiac ischemic tolerance [380]. Such effects might be relevant to observations of acute protection early in STZ-dependent hyperglycemia. Indeed, Jensen et al. [382] present evidence *O*-GlcNAc signaling participates in remote ischemic preconditioning and activates cardioprotection in DM myocardium from T2DM patients (based on functional I–R tolerance of atrial trabeculae).

Other evidence indicates chronic elevations in *O*-GlcNAc are detrimental to the heart. Hyperglycemia mediated HBP activation increases cardiomyoblast death [383], and inhibitors of OGA have been shown to improve I–R tolerance in DM hearts, potentially via preserved integrity of *O*-GlcNAc associated Z-line protein structures [380]. Activation of the HBP and protein *O*-GlcNAcylation modulates hypertrophic and cell signaling pathways in T2DM [378]. Increased protein *O*-GlcNAcylation in non-DM cardiomyocytes also decreased hypertrophic signaling responses, while HBP inhibition partly restored hypertrophic signaling in DM cardiomyocytes. Cardiac beclin-1 and Bcl-2 have also been recently identified as targets for *O*-GlcNAcylation [384], with blunted autophagy in cardiomyocytes from T2DM *db/db* mice partly reversed by inhibiting the HBP. Ramirez-Correa et al. [385] present evidence that Z-line localization of *O*-GlcNAc and OGT and A-band localization of OGA is disrupted, consistent with changes in human DM hearts. Their data indicate subcellular redistribution of OGT and OGA rather than changes in overall activities are responsible for altered *O*-GlcNAcylation in DM. On the other hand, Dassanayaka et al. [386] show *O*-GlcNAcylation is not involved in inhibition of mitochondrial metabolism in hyperglycemic cardiomyocytes.

There is only limited evidence for glycosylation modifications of plasma membrane proteins. In coronary endothelium OGA expression is decreased and OGT expression and *O*-GlcNAcylation increased with DM [387], with CX40 identified as a potential target of *O*-GlcNAcylation regulating cell function. Effects of glucosamine and OGT blockade on post-ischemic Ca^2+^ levels also implicate modulation of sarcolemmal channels [388]. Further studies of cardiac sarcolemmal targets of *O*-GlcNAcylation are required to clarify the role of this process in membrane changes and dysfunction in T1 and T2DM.

#### Palmitoylation

Reversible *S*-palmitoylation (thioester attachment of palmitic acid to cysteine) is an important protein ‘sorting’ signal, governing trafficking and membrane localization [389]. Palmitoylation enhances membrane affinity of many proteins to facilitate membrane accumulation [390]. *N*-myristoylation (amide bond attachment of myristoyl group to N-terminal glycine residues) may also facilitate protein localization to membrane palmitoylases [390, 391]. Within the membrane, palmitoylated proteins have high affinities for cholesterol and sphingolipid-rich domains [392], which is important in targeting proteins to membrane raft regions [393]. Some GPCRs are palmitoylated down-stream of the 7th transmembrane domain [394], which may be required for efficient plasma membrane delivery [395, 396]. Palmitoylation may regulate internalization of some GPCRs and promote trafficking of internalized proteins to the plasma membrane. Ion channel and exchanger functions are also modified with palmitoylation. For example, the cardiac Na^+^/K^+^-ATPase is targeted by palmitoylation, though functional outcomes await detailed study [369]. The inactivation of the Na^+^/Ca^2+^ exchanger is also strongly dependent on palmitoylation [370].

Recent data support induction of endocytosis via membrane protein palmitoylation. Massive endocytosis (MEND) is an adapter-independent form of endocytosis that can rapidly internalize up to 70% of the plasma membrane in response to stressors such as Ca^2+^ overload [397]. Increased plasma membrane palmitoylation promotes MEND in response to mitochondrial stress [398], likely due to clustering of palmitoylated proteins in lipid-ordered domains as a result of palmitoyl chain affinity for the ordered lipid environment [399]. Reilly et al. [370] show that elevations in palmitoylated Na^+^/Ca^2+^ exchanger 1 protein in the plasma membrane accelerates MEND, mirroring effects of palmitoylated phospholemman [397, 398] and suggesting palmitoylated proteins promote formation of lipid-protein domains to trigger endocytosis. Since acyl groups of palmitoylated proteins insert more readily between the phospholipid head groups of curved rather than planar membrane regions [400], palmitoylated proteins will cluster in invaginated lipid-ordered domains that may include caveolae [401], the curved domains formed in endocytosis, and potentially curved junctions between T-tubule and surface sarcolemma. Clustering of palmitoylated membrane proteins with large cytoplasmic domains (e.g. Na^+^/K^+^-ATPase, Na^+^/Ca^2+^ exchanger) may itself promote membrane curvature [402]. Unfortunately, despite such effects and the importance of palmitoylation to sarcolemmal protein trafficking and function, few studies have examined potential roles of altered palmitoylation in the cardiac abnormalities of DM.

#### Membrane oxidation

It is well established that oxidative stress is involved in development and progression of DM and its organ-specific complications [10, 403, 404], and shifts in cardiac stress responses may involve oxidative modification of sarcolemmal elements. Oxidative stress may underlie changes in caveolae and caveolins: Su et al. [275] show the anti-oxidant *N*-acetylcysteine (NAC) limits changes in caveolin-3 together with phosphorylated eNOS known to localize to caveolae. Diabetes reduces association of caveolin-3 and eNOS in cardiomyocytes, an effect countered by antioxidant treatment. Protective effects of NAC on hyperglycemic and hypoxic cell injury were also abolished by knockdown of either caveolin-3 or eNOS, supporting the notion hyperglycemic inhibition of eNOS results from impaired caveolin-3 expression. Membrane lipid metabolism also contributes to oxidative stress: lipoxygenases oxidatively metabolize AA released from the plasma membrane following PC hydrolysis, generating ROS in the process. Hyperglycemia-induced activation of 12/15-lipoxygenase is associated with cardiac oxidative stress and DM cardiomyopathy [404]. However, beyond largely indirect evidence (e.g. preventing caveolar changes with anti-oxidant intervention), there is relatively little information available regarding the specific sarcolemmal targets of oxidative modification in DM hearts, and their roles in associated ischemic intolerance. As for glycation/glycosylation and palmitoylation, further studies are needed to clarify modifications to sarcolemmal proteins in T1 and T2DM, and their roles in altered stress responses.

### Remodeling of the T-tubule system in DM

Despite limited studies, and none in human cardiomyocytes, evidence supports significant remodeling of T-tubules in DM. Studies in skeletal muscle confirm the T-tubule system is a functionally important target governing glucose handling [336, 405, 406]. In heart, McGrath et al. [160] report a pronounced fall in functionally intact SR/T-tubular junctions together with an increased T-tubule area (longitudinal rather than transverse orientation) in rat models of T2DM. A subsequent study in *db/db* mice identified a fall in T-tubule density in this model of T2DM [161]. Despite differing morphological outcomes, both studies highlight diabetic disruption of T-tubule organization and functionality, likely perturbing E–C coupling and contractile function. For example, the synchrony of cardiomyocyte Ca^2+^ release (influencing contractile function and arrhythmogenesis) depends on T-tubule integrity, and disorganization underlies cardiac dyssynchrony in different settings [407–409]. Disruption of T-tubule structure and function may thus mediate the reduction in synchrony observed in DM [161, 410].

Changes specifically within the T-tubule system may also be important in altered substrate handling. Magnetic resonance spectroscopic [411] and biochemical analyses [412] confirm defective GLUT4 translocation in muscle of T2DM patients, while studies in animal models confirm impaired translocation in skeletal [405] and cardiac tissue [413, 414]. Dissociation of T-tubules has been shown to reduce basal and abolish insulin-dependent glucose transport in skeletal muscle [415], confirming a critical role in glucose metabolism and homeostasis. Since the majority of GLUT4 translocation occurs specifically within T-tubules [415, 416] and cholesterol-rich microdomains [253], T-tubule disruption and changes in cholesterol will modify insulin-stimulated GLUT4 exocytosis in DM.

Changes in both membrane cholesterol and caveolae/caveolins may contribute to the T-tubule dysfunction in DM. In skeletal myocytes, cholesterol is more concentrated within T-tubules compared to surface membrane regions [417–419], which may contribute to lower fluidity in the lipid phase of T-tubules compared with most cell membranes [420]. While data are lacking for cardiomyocytes, similar compartmentation in T-tubules is likely. Cardiac Ca^2+^ levels and contractility are sensitive to membrane cholesterol [267, 332], and Zhu et al. [330] recently confirmed cholesterols importance to cardiomyocyte T-tubule stability and E–C coupling, an effect apparently independent of caveolin-3/caveolae. The integrity of intercalated disks and intercellular communication were also sensitive to cholesterol. Caveolae and caveolins are also important in T-tubule development and maintenance of functional integrity [421], including the co-localization and interaction between junctophilin-2 and caveolin-3 in dyadic structures to establish efficient, synchronous EC coupling. Depletion of caveolin-3 could contribute to loss of dyadic integrity, and junctophilin/caveolin-3 interactions are known to be suppressed in cardiomyopathy [422].

### Changes in gap junctions and sarcolemmal ion channels in DM

#### Gap junctions

Abnormal conduction and arrhythmogenesis is evident in both DM patients [423, 424] and animal models of T1 and T2DM [425–427]. Together with changes to the T-tubule system, shifts in gap junctions [162] and sarcolemmal ion (Ca^2+^, Na^+^, K^+^) channels will disrupt electrophysiology in DM, and influence cardiac responses to I–R. Specialized gap junction pores provide effective electrical coupling of adjacent cardiomyocytes, and are critical not only to conduction and electrical stability but responses to ischemia and cardioprotective stimuli [428]. Principle connexin (CX) protein components are altered in DM, including evidence of modified expression and phosphorylation. The latter post-translational changes are functionally important: PKC inhibits cardiac gap junction conductance [429, 430] via CX-43 phosphorylation [431, 432]; dephosphorylation of gap junction elements results in their uncoupling [433] and lateralization [432, 434]; and excess phosphorylation of CX-43 by PKCε may promote proteolysis to deplete junction channels in DM myocardium [435].

In cultured myocytes CX-43 expression is suppressed by hyperglycemia [436], potentially involving PKC-dependent miR-1/206 expression [437]; and by the AGE-RAGE system, potentially involving PKC and ERK signaling [438]. In STZ-dependent T1DM in rats an increased SA nodal expression of CX-43 (and -40 and -45) is associated with nodal conduction delay [439], while ventricular expression is reportedly unaltered [440], reduced [435] or increased [441, 442]. Olsen et al. [426] observe reduced lateralization of CX-43 in hearts from ZDF rats exhibiting reduced conduction velocity. Phosphorylation of atrial and ventricular CX-43 declines in models of DM [439, 442, 443], potentially as a result of impaired PKCε expression [444], though these investigators also report increased PKCε mediated CX-43 phosphorylation in DM myocardium, which may promote proteolytic degradation [435]. The extent of cardiac CX-43 phosphorylation reportedly declines with progression of DM while protein nitration increases [443]. Zhu et al. [330] also recently found that cholesterol depletion not only destabilized cardiomyocyte T-tubules, but disrupted the integrity of intercalated disks and intercellular communication. Gap junction function and inter-cellular communication may therefore be influenced by sarcolemmal cholesterol changes in DM. Supporting mechanistic involvement of gap junction changes in the myocardial abnormalities of DM, benefit with exercise in T2DM *db/db* mice is attributed to restoration of CX-43 networks [445], and beneficial effects of n-3 PUFA feeding on DM cardiomyopathy are linked to increased CX-43 expression and phosphorylation (associated with up-regulated PKCε) [414]. In contrast, it has also been reported that moderate exercise reduces ventricular CX-43 phosphorylation [446].

#### Ion channels

Sarcolemmal ion channels fundamental to E–C coupling and relevant to I–R injury are modified in DM myocardium, including changes in Ca^2+^ channels, levels and contractile responses [447–451], K^+^ currents and channels [452–454], and Na^+^ pumps [247, 455]. These may participate in enhanced arrhythmogenesis and risk of sudden cardiac death [423, 425, 427, 452]. Altered membrane lipids and biophysical properties in DM will influence ion channel function, and changes in channel transcription and expression patterns also arise. There is evidence of increased transcription of Ca^2+^ channels (*Cacna1c, Cacna1g, Cacnb1*) and *Gja4* (CX-37), and differential changes in K^+^ channels (*Kcnj11* up, *Kcnb1* down) in GK T2DM rat hearts [450]. Sucrose feeding induces K^+^ channels (*Kcnj2, Kcnj8*) and *Gja1* (CX-43) and *Gja4* in non-DM rats [450]. This group also reports up-regulation of ventricular *Cacna1h, Scn1b* and *Hcn2* vs. down-regulation of *Hcn4, Kcna2, Kcna4* and *Kcnj2* in this model [449], and up-regulation of genes encoding cardiac LTCC proteins (*Cacna1c, Cacna1g, Cacna1h* and *Cacna2d1*) in association with prolongation of Ca^2+^ transients in the ZDF rat model of T2DM [448].

These transcriptional changes do translate to altered channel expression, with shifts in Ca^2+^, K^+^ and Na^+^ channels all potentially contributing to electrophysiological perturbations in DM hearts. Abnormal Ca^2+^ currents in cardiomyocytes from T1DM Akita mice involve reduced sarcolemmal levels of the LTCC, potentially related to impaired PI3K control [456]. The decline in sarcolemmal Ca^2+^ permeability in T2DM *db/db* mice is associated with reduced expression of the pore-forming α1C subunit of the LTCC [447]. Though less well studied, cardiac T-type Ca^2+^ channel expression/function may also be modified in DM given caveolar localization and sensitivity to caveolin-3 [457], and evidence of changes in other cell types with chronic DM [458]. Reductions in cardiomyocyte K^+^ current density in models of T1DM also involve defective channel expression (potentially involving AMPK signaling) [459], and action potential prolongation in Otsuka-Long-Evans-Tokushima Fatty rats is linked to down-regulation of endocardial Kv4.2 (voltage-gated K^+^ channel subfamily D) and transmural KChIP2 (K^+^ channel interacting protein) expression [454]. Impaired insulin signaling has been shown to reduce the amplitude of the transient outward K^+^ current fast component in cardiomyocytes in association with reduced Kv4.2 and KChIP2 expression [453]. Broadened ventricular repolarization and reduced ‘repolarization reserve’ in alloxan-induced T1DM in dogs may also involve impaired K^+^ currents as a result of reduced Kv4.3 (voltage-gated K^+^ channel subfamily D) and MinK (voltage-gated K^+^ channel sub-family E subunit) expression, while Kv1.4 (voltage-gated K^+^ channel subfamily A), KChIP2 and KvLQT1 (voltage-gated K^+^ channel subfamily D) were increased [452]. Such changes may significantly predispose to sudden cardiac death.

Depressed *I*_Na_ may additionally play a role in altered electrical activity in DM cardiomyocytes, with less Na^+^ influx during contraction linked to reduced expression of both the Na^+^/K^+^-ATPase and Na^+^/Ca^2+^ exchanger [247]. This is consistent with sarcolemmal Na^+^-K^+^ pump inhibition in other models of T1DM [455]. Changes in Ca^2+^, K^+^, and Na^+^ channels are not only likely to increase susceptibility to arrhythmias in I–R, but may well modulate cell death processes. Moreover, expression of subunits for the sarcolemmal K_ATP_ channel implicated in multiple cardioprotective responses [72] is also disrupted in DM, with SUR2A and Kir6.2 decreased both in myocytes from T1DM rats and isolated myocytes subjected to hyperglycemia [460]. Such a change will not only desensitize cardiac myocytes to K_ATP_ openers, but impair transduction of cardioprotective signaling. The impacts of these varied channel expression changes in DM will be exacerbated by membrane lipid changes and structural modifications to the sarcolemma, including shifts in microdomains in which select channels cluster, and T-tubule and gap-junction remodeling.

## Potential ‘membrane-targeted’ therapies?

Based on the array of detrimental sarcolemmal changes evident in DM, a number of therapeutic approaches present themselves, including modifications to diet and physical activity, cholesterol manipulation, and modulation of caveolins and caveolar biology.

### Targeting caveolae and caveolins

Given evidence of abnormal caveolin-3 expression in models of DM, this caveolar protein has appeal as a therapeutic target [158, 159, 169]. Beneficial anti-diabetic effects of hepatic caveolin-3 gene transfer supports the therapeutic potential of caveolin-3 in DM [315]. Although the regulation of cardiac caveolin-3 expression is not well understood, there is evidence from non-cardiac cells for transcriptional control by myogenin, ID2, miR-22 and myocardin [289]. Myocardins are important to formation of caveolae, and in glucose and lipid homeostasis [292]. Whether transcriptional control of caveolins might be targetable is not clear. However, hyperinsulinemia does up-regulate myocardin in cardiac myoblasts [290], which additional to modulating hypertrophy could up-regulate caveolins and caveolae [289]. Conversely, insulin-resistance may reduce myocardin expression and thereby caveolins and caveolae. Aortic myocardin is substantially induced in GK (T2DM) rats, which appears to involve a miR-145 dependent response to oxidative stress [291].

Interestingly, cardiac caveolin-3 may be differentially modifiable via dietary saturated fat [276] and PUFA [278, 461], and hyperglycemic depression of caveolin-3 may also be PKCβ2-dependent, providing a potential pharmacological target. Lei et al. [240] show that inhibition or knockdown of PKCβ2 counters hyperglycemic depression of caveolin-3 in hearts and myocytes, and improves cardiac Akt phosphorylation and diastolic function. This group subsequently showed that PKCβ2 inhibition also improved cardiac I–R tolerance together with caveolin-3 levels and control of Akt signaling in STZ-dependent T1DM rats [241]. Supplementation with the anti-oxidant NAC also attenuates PKCβ2 expression and hypertrophy [462] while enhancing ischemic tolerance [275] in STZ-dependent T1DM rats. Caveolin-3 levels were not measured, though a reduction in PKCβ2 activity is predicted to improve caveolin-3 based on other work [250].

Other potential targets include adenylyl cyclase (AC) and focal adhesion kinase (FAK) signaling paths: in vitro studies suggest AC can repress caveolin-3 in cardiac myoblasts [463] while FAK up-regulates caveolin-3 in skeletal myoblasts [464]. No data are available regarding cardiac FAK signaling in DM, however, FAK may be activated in hyperglycemic conditions [465], and FAK induction in hypertrophied skeletal muscle is exaggerated in T1DM rats [466]. In skeletal myotubes FAK also appears important in insulin-dependent GLUT4 translocation and glucose uptake [467]. Adenylate cyclase itself appears functionally unaltered in DM hearts, while adrenergic receptor mediated control is impaired [468]. However, vascular AC expression/function may be altered in DM [469]. Inhibition of cardiac AC5 activity does protect against cardiac abnormalities in T2DM and obesity [470].

Targeting caveolin-3 expression via acute gene therapy with adeno-associated virus (AAV) for *Cav3* improves I–R and Ca^2+^ tolerance, preserves mitochondrial stability and reduces reactive oxygen species [281]. In addition, cardiac specific caveolin-3 overexpression enhances functional outcomes post-I–R and reduces infarct size (similar to effects of ischemic preconditioning), which may be due to improved mitochondrial Ca^2+^ tolerance and respiratory rates with reduced ROS generation [281]. Fridolfsson et al. [281] identified that increased O_2_ consumption in caveolin-3 overexpressing hearts improved energy production without a parallel increase in ROS generation. Further experiments targeting caveolin-3 to mitochondria confirmed improved mitochondrial stability during Ca^2+^ challenge, and delayed mitochondrial depolarization and improved respiratory complex activity associated with enhanced ischemic tolerance. Conversely, deletion results in mitochondrial dysfunction [281] and hypertrophy [287, 288]. How caveolin-3 migrates to/communicates with mitochondria and subsequently promotes mitochondrial and ischemic tolerance remains to be further detailed.

### Cholesterol lowering therapies

Reducing membrane cholesterol has capacity to improve fluidity and counter some sarcolemmal abnormalities evident in DM hearts. On the other hand, whether reductions in cholesterol might adversely impact caveolae, caveolins and T-tubules is unclear. Certainly, statins are of value in DM, with low-dose treatment significantly reducing cardiovascular events in T2DM patients [471]. However, while pleiotropic effects of statins include ‘anti-diabetic’ actions such as reduced inflammation in T2DM patients [472], they may also include induction of insulin-resistance and promotion of DM [473, 474]. That said, such effects appear modest relative to the benefits of statins, and may only be a factor in those at particular risk of new onset DM [475]. Experimental studies show statins do protect against myocardial ischemic injury in hearts from DM and healthy animals, though again this reflects pleiotropic effects of the drugs independent of cholesterol lowering [476, 477].

### Dietary intervention

Modifiable diet and physical activity have long been appreciated as major determinants of DM severity and complications. Dietary modification can alter sarcolemmal makeup and function, and inflammatory, glycation/glycosylation and oxidative processes in the heart and vessels. For example, homeostatic control of inflammation is mediated by eicosanoids (prostaglandins, leukotrienes, thromboxanes) whose generation is dependent on the n-6 PUFA AA [179]. Shifts in saturated vs. unsaturated fat intake can modify fundamental membrane properties together with caveolar components, while limitations in caloric intake may profoundly influence the DM phenotype and promote protective outcomes.

#### Unsaturated vs. saturated fats

Mammalian species are unable to produce n-3 PUFAs, thus must acquire these essential fatty acids via the diet. Edible seeds such as flaxseed and chia seeds are rich sources of the 18C n-3 PUFA α-linolenic acid, while longer chain n-3 PUFAs (EPA, DHA) can be synthesized from α-linolenic acid or consumption of fish oils. Once acquired, n-3 PUFAs can integrate into the sarcolemma to displace membrane AA: dietary n-3 PUFA incorporation in myocardium and myocytes occurs at the expense of n-6 PUFAs [478]. Consumption of n-3 PUFAs thus reduces inflammation via disrupting production of AA-derived eicosanoids [479, 480]. However, it is worth noting that AA-derived eicosanoids (including prostaglandin E2) exhibit both pro- and anti-inflammatory capabilities.

Dietary α-linolenic acid is cardioprotective in a rat model of T2D [143], with 4 weeks of α-linolenic acid supplementation improving ischemic tolerance including enhanced functional outcomes and reductions in infarction and markers of cell death (whereas no protection was evident in non-DM rats). Cardioprotection was linked to anti-inflammatory (reduced tumor necrosis factor-α and interleukin-6) and anti-oxidative (reduced superoxide and enhanced anti-oxidant capacity) actions, possibly involving PI3K/Akt signaling [143]. Insulin-resistance, glucose intolerance, dyslipidemia and cardiac lipid accumulation after 3–6 months of a high-sugar diet are also reversed by transition to a chia seed-rich diet [481]. Consumption of n-3 PUFAs improves sarcolemmal functions, critical to the management of DM cardiomyopathy. For example, consumption of fish oils: enhances EPA and DHA in cardiac membranes while reducing AA [479, 480]; prevents translocation of CD36, limiting fatty acid uptake and lipid storage [482]; and counters abnormal membrane fluidity in T1DM mice [195]. A vegetarian diet improvement in linoleic acid content is also associated with improved insulin sensitivity in subjects with T2DM [483].

Diets containing high ratios of PUFA/mono-unsaturated fatty acid (MUFA) improve insulin-binding and glucose uptake in adipose cells from healthy and T1DM rats [484]. Membranous phospholipid content is also altered, with enhanced PUFA and reduced MUFA (though no effect on total saturated phospholipids) [484]. Interestingly, even at very high insulin levels (1000 ng/mL), cells from T1DM rats fed low PUFA/MUFA diets bind less insulin than those fed high PUFA/MUFA diets and exposed to lower insulin levels. This suggests that insulin has greater affinity for cells with more unsaturated membranes, which may be particularly useful in management of insulin-resistant T2DM.

Enriched n-3 PUFA diets also modify ion exchange and action potential duration, which may limit cardiac propensity to I–R injury and arrhythmias. Isolated myocytes from rabbits fed fish oil for 3 weeks exhibit increased sarcolemmal EPA and DHA (vs. decreased MUFAs) and 20% shorter action potentials compared with myocytes from animals on a n-9 MUFA-rich diet [485]. Exposure of myocytes to EPA and DHA shortened action potentials in cells from n-9 MUFA and not n-3 PUFA fed rabbits. These findings indicate action potential shortening likely stems from altered membrane lipid composition and not direct ligand-like interaction with ion channels [485]. Other studies report inhibitory effects of PUFAs on sarcolemmal K^+^ [486] and Ca^2+^ channels [487], and the Na^+^/H^+^ exchanger [488], potentially limiting pathological Ca^2+^ overload in myocardial cells.

Dietary fats also influence caveolin expression and thus caveolae. A palmitate enriched diet significantly depresses cardiac caveolin-3 [276], whereas a flaxseed-enriched diet reverses reductions in cardiac caveolin-3 in cardiomyopathic hamsters [278], and prevents reductions in skeletal muscle caveolin-3 in a model of muscular dystrophy (also repairing sarcolemmal damage, reducing inflammation and cell death) [459]. Effectiveness of such diet intervention in a DM animal model awaits testing. In addition to n-3 PUFA supplementation, improved cardiac function in DM may be achievable through calorie restriction (CR).

#### Caloric limitation and time-restricted feeding

Calorie restriction or intermittent fasting may provide significant benefit in DM, and such interventions modify membrane composition in murine myocardium [489]. Though prolonged caloric limitation is a well-established protective stimulus, effects of brief or moderate fasting await detailed study in DM animals. Severe CR for 11 days generates unique I–R tolerance [490], and 24–72 h of fasting enhances cardiac I–R tolerance and mitochondrial viability in non-DM hearts [491, 492]. There are surprisingly few studies of caloric limitation in DM. A 30% limitation in calories for ≥ 2 months improves glucose homeostasis and markers of systemic or cardiac oxidative-stress in rodent models of T2DM [493, 494]. A similar CR regime fails to influence I–R tolerance in models of T2DM and metabolic syndrome, though benefit via ischemic preconditioning was restored [144]. Contrasting reported protection with fasting, one recent study suggests 18 h of fasting actually worsens ischemic tolerance in T2DM and also non-DM rat hearts [495], potentially linked to enhanced glucose vs. fatty acid metabolism. Another recent study [496] also found that loss of sevoflurane preconditioning with a high calorie western diet was unaltered with 4 week of control diet (though an apparently detrimental impact of sevoflurane with the western diet was countered).

Whether ischemic tolerance with caloric limitation involves membrane changes in either DM or non-DM hearts remains to be established. However, modest (12 h) fasting does induce membrane remodeling via a reduction in acyl chains, predominately lost from C22:6 (DHA) species [489]. While effects of CR on myocardial caveolar domains are unknown, it does prevent age-related reductions in caveolin-1 in liver tissue [497], and repression of caveolin-1 in breast tissue is mediated by a micro-RNA (miR-203) that is induced with CR [498].

Circadian biology is extremely important in the influences of fat and calorie intake on obesity and associated metabolic disturbances, and restricted feeding times rather than calorie intakes can be highly beneficial in cardiometabolic disorders [499]. The timing of food intake appears a key determinant of circadian rhythm, particularly in metabolic organs, and the impacts of high-fat feeding on body weight, insulin levels, glucose tolerance, inflammation and hepatic steatosis can be effectively countered by time-restricted feeding without caloric limitation [500]. Conversely, short-term feeding at the wrong time of day can desynchronize peripheral clocks and induce obesity and metabolic disorder [501]. Time-restricted feeding also counters cardiac aging changes in the Drosophila model [502], however effects on myocardial ischemic tolerance, or the cardiomyopathy and sarcolemmal changes in DM, have yet to be tested.

### Exercise in DM—membrane involvement?

Physical activity and VO_2_ have been identified as perhaps the most important factors governing chronic disease risk, particularly CVD and DM [503]. Up to 50% of coronary artery disease can be prevented by 30 min of moderate exercise daily (assessed in middle-aged women) [504–506], and as little as 3 weeks of exercise can reduce the clinical impact of metabolic syndrome (a combination of coronary heart disease, hypertension and T2DM) by 50% [507]. Not only substantially reducing risk/incidence, exercise can be applied ‘therapeutically’ in existing disease states to alleviate symptoms and counter progression. Broadly beneficial systemic effects render physical activity an effective therapy in disorders including cancer [508], depression [509] and cardiovascular disease [510]. Exercise induces obvious metabolic advantages, improving tissue and whole body VO_2_/oxidative capacity and vascularity, cardiac functional reserve and efficiency, insulin signaling and sensitivity, glucose and fat handling, anti-oxidant status, inflammation and immune function [510–512]. Analyses confirm benefits of physical activity in patients with T2DM, though questions regarding effective exercise prescription remain [513, 514]. Not only reducing the incidence of infarction, exercise also boosts myocardial tolerance to infarction [515] and may improve or restore conventional protective responses in models of stress, disease and aging [516]. Effects on the DM heart revolve around improved glucose and fatty acid metabolism, mitochondrial function and oxidative stress, however sarcolemmal abnormalities are also influenced. Exercise does modify fatty acid composition of phospholipids and triglycerides in cardiac and skeletal muscle [517, 518], and beneficial remodeling of plasma membrane lipids is reported in other cell types [519]. Sarcolemmal effects in DM are less well defined.

Studies confirm exercise-dependent improvements in cardiac function, survival signaling and ischemic tolerance in models of T1DM [136] and T2DM [146, 520, 521]. While improvements in substrate metabolism are broadly implicated, Pons et al. [520] report cardioprotection in *ob/ob* mice independent of hyperglycemia, hypercholesterolemia, hyperinsulinemia, fat mass or body weight. Schrauwen-Hinderling et al. [522] found that 12 week endurance/strength training improves systemic insulin sensitivity and cardiac function in T2DM patients without modifying cardiac lipid content. Altered myocardial *O*-GlcNAcylation may participate, with evidence swimming in T1DM rats increases OGA activity and reduces cardiac protein *O*-GlcNAcylation [523]. However, this also reduces *O*-GlcNAcylation in non-DM hearts [524]. Indeed, Medford et al. [525] show as little as 15 min of exercise can alter myocardial *O*-GlcNAcylation. Exercise protection in models of DM has been linked to normalization of nitro-oxidative stress and eNOS control [146], and improvements in PPARγ coactivator-1α and Akt signaling [521], both effects that may arise via restoration of sarcolemmal caveolae and caveolin control of eNOS [250, 275, 296–298] and Akt signaling [250–252, 299]. Studies in non-DM [526] and DM hearts [294] do support up-regulation of caveolin-3, though the contribution of this change to exercise cardioprotection awaits analysis. Indirectly supporting targeting of sarcolemmal elements, da Silva et al. [449] show that altered Ca^2+^ transients (and mitochondrial uptake) in T1DM rat hearts are countered by swimming, which also enhanced benefit via insulin.

More directly supporting improved sarcolemmal makeup, Hesari et al. [444] report that exercise reduces CX-43 phosphorylation in hearts from T1DM rats, and Veeranki et al. [443] demonstrate beneficial effects of exercise on CX-43 levels and gap-junction function in *db/db* mice, associated with preservation of mitochondrial function. This is consistent with evidence exercise modulates sarcolemmal determinants of signaling and E–C coupling in T2DM rats, including transcriptional up-regulation of caveolin-3 and CX-43, and differential changes in K^+^ channels (*Hcn2, Kcnk3*) [294]. Our unpublished findings support up-regulation of cardiac caveolin-3 and protection against I–R with swim training in mice, coupled with powerful anti-inflammatory effects of exercise (data not shown).

## Conclusions and perspectives

A diversity of mechanisms are involved in the cardiac and coronary abnormalities arising in DM, and evolution of DM cardiomyopathy. However, the sarcolemma is a nexus for many fundamental mechanistic elements and sequelae of DM. The ability of the sarcolemma to withstand rupture is fundamentally important to cell survival and stress tolerance and is governed by molecular makeup and caveolar membrane ‘reserve’. The sarcolemma is also the seat of glucose and fatty acid transport and InsR control, and therefore fundamentally participates in the pathogenesis of DM complications. Furthermore, the functionality of ion channels and cell surface receptors is determined by membrane makeup. Diabetes impacts sarcolemmal architecture, remodeling T-tubules, caveolar domains and gap junctions, disrupting E–C coupling and promoting injury and arrhythmogenesis in I–R. Specific molecular changes include increased cholesterol and fatty acid saturation vs. reduced desaturation, and differential shifts in phospholipids and PUFAs. Caveolar proteins are a particularly important target in DM, with evidence for caveolin-3 depletion and caveolae dysfunction in dysregulation of GLUT4 and CD36 function, survival kinase and eNOS signaling. Importantly, the sarcolemma is malleable, responsive to dietary modification, physical activity and other interventions. A further unraveling of the roles of sarcolemmal changes in DM and its cardiac complications thus has potential to inform approaches to managing these disorders, improving ischemic tolerance and developing cardioprotective therapies for the DM population. This requires further focused investigation of sarcolemmal changes in animal models and particularly in sufferers of T1 and T2DM, though the latter presents a significant experimental challenge.
